# Small-residue packing motifs modulate the structure and function of a minimal de novo membrane protein

**DOI:** 10.1038/s41598-020-71585-8

**Published:** 2020-09-16

**Authors:** Paul Curnow, Benjamin J. Hardy, Virginie Dufour, Christopher J. Arthur, Richard Stenner, Lorna R. Hodgson, Paul Verkade, Christopher Williams, Deborah K. Shoemark, Richard B. Sessions, Matthew P. Crump, Michael R. Jones, J. L. Ross Anderson

**Affiliations:** 1 School of Biochemistry, University of Bristol, Bristol, UK; 2 School of Chemistry, University of Bristol, Bristol, UK; 3 BrisSynBio, Life Sciences Building, Tyndall Avenue, Bristol, BS8 1TQ UK

**Keywords:** Membrane proteins, Biophysics, Membrane structure and assembly

## Abstract

Alpha-helical integral membrane proteins contain conserved sequence motifs that are known to be important in helix packing. These motifs are a promising starting point for the construction of artificial proteins, but their potential has not yet been fully explored. Here, we study the impact of introducing a common natural helix packing motif to the transmembrane domain of a genetically-encoded and structurally dynamic de novo membrane protein. The resulting construct is an artificial four-helix bundle with lipophilic regions that are defined only by the amino acids L, G, S, A and W. This minimal proto-protein could be recombinantly expressed by diverse prokaryotic and eukaryotic hosts and was found to co-sediment with cellular membranes. The protein could be extracted and purified in surfactant micelles and was monodisperse and stable in vitro, with sufficient structural definition to support the rapid binding of a heme cofactor. The reduction in conformational diversity imposed by this design also enhances the nascent peroxidase activity of the protein-heme complex. Unexpectedly, strains of *Escherichia coli* expressing this artificial protein specifically accumulated zinc protoporphyrin IX, a rare cofactor that is not used by natural metalloenzymes. Our results demonstrate that simple sequence motifs can rigidify elementary membrane proteins, and that orthogonal artificial membrane proteins can influence the cofactor repertoire of a living cell. These findings have implications for rational protein design and synthetic biology.

## Introduction

Integral membrane proteins are ubiquitous and essential in biology. Alongside continuing efforts to understand natural membrane proteins, there is now an emerging interest in designing artificial membrane proteins from first principles. Such de novo proteins can help reveal the fundamental relationships between primary sequence, structure, and function^1,2^. They are also important new research tools to examine the role of complexity in protein chemistry^3–6^, to survey the scope and accessibility of membrane protein structural space^7–9^, and could be the basis of novel enzymes^10–12^. Realising the potential of such artificial proteins requires the elucidation of core design elements that can support their folding and activity.

The majority of natural integral membrane proteins consist of bilayer-spanning alpha-helices connected by soluble domains. A key contribution to the folding and assembly of these proteins comes from van der Waals interactions between neighbouring transmembrane helices^13^. These forces are optimised in natural proteins through a relatively limited number of sequence motifs that minimise the interhelical distance through sidechain packing. A bioinformatic survey^14^ revealed that nearly one-third of natural transmembrane helical pairs pack with a slight left-handed crossing angle via an antiparallel Ala-coil-like motif. This motif was termed GAS_Left_ to reflect the frequency of small sidechains (G, A or S) at the *a* and the *d* or *e* positions of the helical heptad. The second most abundant packing code among helical pairs was termed GAS_Right_. This was essentially the Small-xxx-Small motif^15–17^ that was also identified, along with several other patterns, as underlying the packing of helical trimers within the membrane^2^. As well as allowing the close approach of neighbouring helices and sidechain burial via knobs-into-holes interactions, the Small-xxx-Small motif enables additional stabilising interactions via interhelical backbone hydrogen bonding^18,19^. Extending this pattern within a transmembrane segment results in the glycine zipper that is found in multiple structural contexts^20^.

The ease with which such motifs can be exploited in protein design remains an open question. Previous examples of de novo membrane proteins have needed to supplement packing interfaces with explicit hydrogen bond networks^8,21,22^, cofactor binding^11^ or metal binding^10^ to help consolidate their structure. It has recently emerged that classical coiled-coil heptads featuring bulky side-chains at the interfacial *a* and *d* positions can produce very well-defined tertiary structures through van der Waals forces alone^7^. However, it remains to be seen whether motifs incorporating small sidechains can be similarly integrated into de novo designs. A key challenge is that helical interfaces based around small residues can lack the favourable steric and energetic effects that arise from the interdigitation of larger groups.

One way to explore this issue is to incorporate small-residue helix packing motifs into model individual transmembrane segments and study their impact on helical association^16,23^. We recently established a new experimental system that could support such studies in the context of a biosynthesised protein. Our approach was based around four-helix bundles of minimal sequence complexity that are genetically-encoded and can be integrated into the membrane of a biological cell^24^. These bundles were given the sobriquet REAMP, for ***r***ecombinantly-***e***xpressed ***a***rtificial ***m***embrane ***p***roteins. The prototypical REAMP design used only the amino acids L, S, G and W to form four transmembrane helices connected in an antiparallel topology by short extramembrane linkers. This REAMP could be purified in a stable and monodisperse state from the cytoplasmic membrane of recombinant *Escherichia* (*E.*) *coli* and rationally engineered to bind redox-active cofactors in vitro. The initial proof-of-principle REAMP sequence did not stipulate any tertiary packing interactions within the transmembrane domain. Unsurprisingly, NMR spectra were consistent with a dynamic molten globule, presumably reflecting the presence of multiple isoenergetic states. The simplicity, tractability and apparent flexibility of this REAMP thus provides a ‘blank slate’ to explore how interhelical interactions can modulate the conformational heterogeneity and rigidity of de novo membrane proteins.

Here, we introduce repeating units of the GAS_Left_ packing motif into the REAMP helices and investigate the effect of these mutations on protein packing and dynamics. We go on to determine the positive impact of this redesign on cofactor binding and protein function and explore how this second-generation REAMP might be assimilated into the biochemistry of a biological cell. Our results suggest that generic sequence motifs can reduce the structural dynamics of artificial membrane proteins, and advance the concept that catalytic membrane proteins are a credible target for de novo design.

## Results

### Protein design

**Figure 1:**
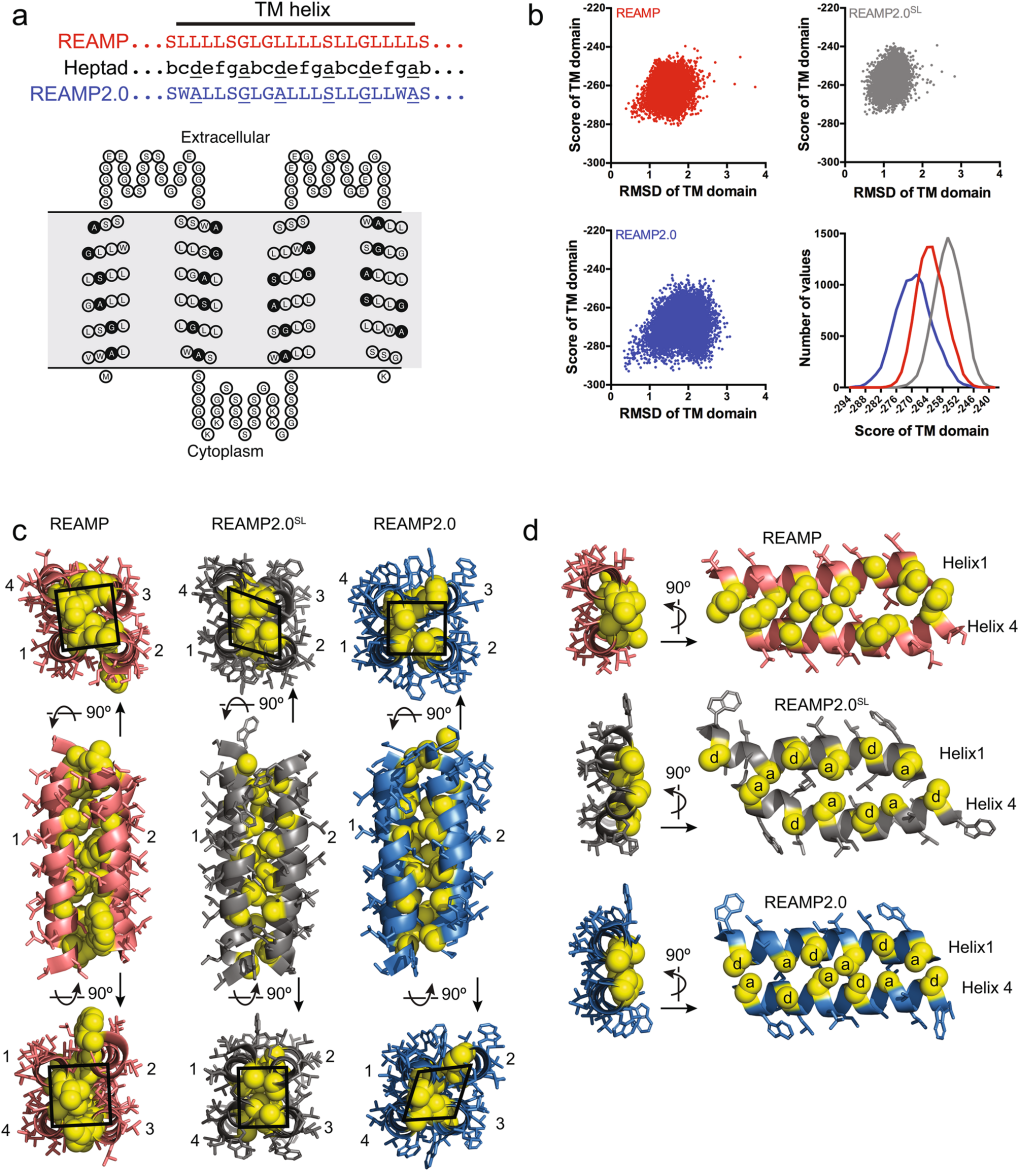
Designing the second-generation de novo membrane protein REAMP2.0. (**a**) Six rational mutations in each transmembrane helix of the prototype REAMP sequence generated a GAS_Left_ packing motif with small residues at the *d* and *a* positions (underlined). Addition of two Trp per helix produced the sequence REAMP2.0. The predicted transmembrane topology is shown (https://www.sacs.ucsf.edu/TOPO2/). (**b**) Output of whole-protein packing simulations with RosettaMP, using 10,000 decoys. RMSD is calculated relative to the decoy with the lowest score for the transmembrane (TM) domain. (**c**) Lowest-scoring structures from packing simulations, with loops omitted for presentation. Residues corresponding to the GAS_Left_ positions are shown as yellow spheres. *SL*, short loops. Geometric shapes show the approximate position of the helix centres and are intended only as a guide to the eye. (**d**) Small residues can allow the close approach of neighbouring helices. The heptad positions *d* and *a* are shown for REAMP2.0.

Inspection of the original REAMP sequence^24^ identified a helical register that could incorporate the GAS_Left_ packing motif using the fewest mutations (Fig. 1a). The resulting second-generation design, termed REAMP2.0, comprised four antiparallel transmembrane segments each with the sequence WALLSGLGALLLSLLGLLWAS (heptad positions *d* and *a* underlined). During this design process we took the opportunity to introduce two Trp residues to each helix, to increase the protein absorption signal and to allow for membrane interface ‘anchoring’ by the amphipathic Trp sidechain^25^. The flexible interhelical loops were also extended from 7 to 26 residues to remove any potential loop constraints on helical mobility. We deliberately avoided any further computational optimisation of the sequence in order to preserve sequence austerity. Additional designs incorporated either one or two histidines to promote cofactor binding at specific locations. These mutations were S15H and S15H/L108H, producing REAMP2.0^H^ and REAMP2.0^H/H^ respectively. Bioinformatic predictions indicated that REAMP2.0 would form a multipass four-helix bundle with the N- and C-termini located in the cytoplasm (Fig. 1a, Supplementary Table 1).

Although ab initio modelling was challenging given the novel and repetitive nature of the REAMP sequences, we built preliminary model proteins based on known helical bundles and used these for packing simulations with RosettaMP (Fig. 1b). To allow direct comparison, the resulting decoys were rescored considering only the transmembrane domain. Although introducing the GAS_Left_ motif and extending the loops did result in a slightly broader distribution of both the Rosetta score and RMSD, REAMP2.0 could access lower-scoring structures than a variant that incorporated the GAS_Left_ motif but left the loops unchanged (REAMP2.0^SL^). The increase in conformational space afforded by the longer loops in REAMP2.0 thus appears to be helpful in reaching optimal packing interactions. Figure 1c shows the decoys with the lowest transmembrane scores in each case. These models showed packing reminiscent of natural GAS_Left_ proteins, with small sidechains found at the helix interfaces (Fig. 1d). All of the low-scoring models (including the original REAMP sequence) had at least two helical pairs that adopted the modest left-handed helix crossing angle characteristic of GAS_Left_. In REAMP2.0 this was helices 1/4 and 2/3 and, with angles close to 178° and 174° respectively. In contrast helix pairs 1/2 and 3/4 had a slight right-handed packing angle of 178° and 174°, respectively.

### Protein expression and purification

**Figure 2:**
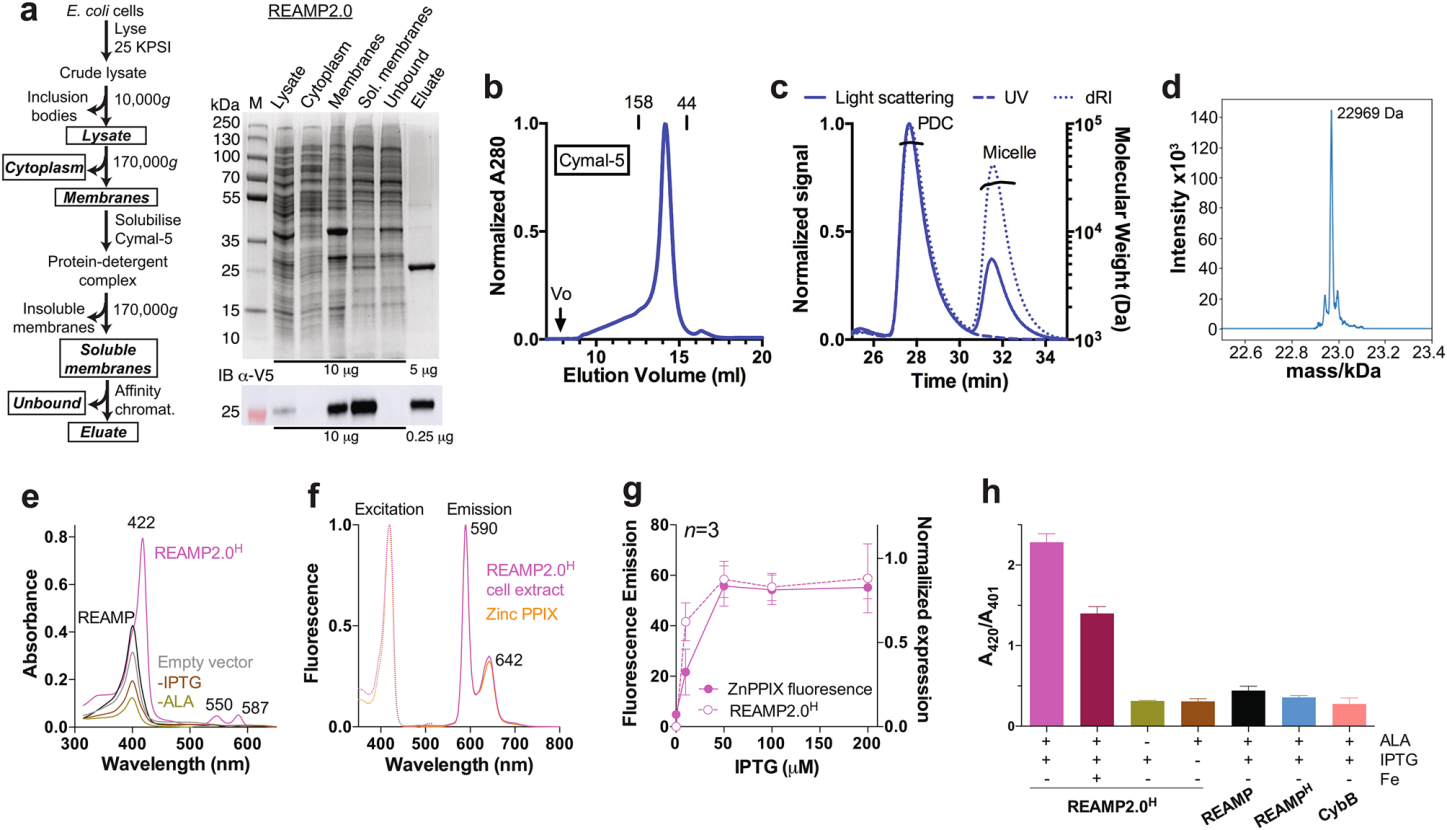
Recombinant expression and purification of REAMP2.0. (**a**) REAMP2.0 was isolated from *E. coli* cellular membranes by affinity chromatography. Cell fractions boxed in the workflow are retained and analysed by Coomassie-stained SDS-PAGE and anti-V5 western blot (*IB a-V5*). The theoretical molecular weight of the *Strep*II-tagged REAMP2.0 is 22.9 kDa. The uncropped western blot is provided as Figure S3. (**b**) Size exclusion chromatography, (**c**) static light scattering and (**d**) native nanoelectrospray mass spectrometry all confirm that purified REAMP2.0 is a homogenous, monodisperse monomer in the solubilising detergent Cymal-5. (**e**) Solvent extracts of cell membranes from induced strains of REAMP2.0^H^ accumulate a novel pigment when supplemented with the heme precursor *δ*-aminolevulinic acid (ALA). Treatment controls shown include uninduced (*-IPTG*) and unsupplemented (*-ALA*) strains. (**f**) fluorescence spectra of membrane extracts confirm the pigment as zinc protoporphyrin IX by reference to a commercial standard. (**g**) REAMP2.0^H^ expression correlates with cellular zinc porphyrin. (**h**) Ratio of absorption peaks from ZnPPIX (A_420_) and heme (A_401_) in membrane solvent extracts. + *Fe*, culture media with 0.1 mM ammonium iron sulfate. *CybB*, strain overexpressing recombinant *E. coli* diheme cytochrome CybB. Data in (**g**) and (**h**) are mean ± SD of 3 independent repeats.

A synthetic gene corresponding to REAMP2.0 (Fig. S1) was recombinantly expressed in *E. coli*. Although most of the expressed protein formed cytoplasmic inclusion bodies (Fig. S2), some REAMP2.0 co-sedimented with cellular membranes (Fig. 2a). This protein could be solubilised in the mild surfactant 5-cyclohexyl-1-pentyl-β-D-maltoside (Cymal-5) and purified by affinity chromatography using either His_10_ or triplet *Strep*II tags at yields of 6 mg (0.3 μmol) REAMP2.0 per g total membrane protein. The *Strep*II tag gave higher purity and so was used for all subsequent experiments. REAMP2.0 in cytoplasmic inclusions could not be solubilised with Cymal-5, implying a fundamental difference between REAMP2.0 in cytoplasmic aggregates and protein associated with cell membranes. Attempts to use covalent labelling to determine whether REAMP2.0 was inserted across the *E. coli* inner membrane were unsuccessful, because introducing Cys mutations into any of the putative extramembrane loops abolished protein expression.

To determine whether REAMP2.0 was compatible with other recombinant hosts, the same gene was also expressed in the purple photosynthetic α-proteobacterium *Rhodobacter sphaeroides* and the model eukaryote *Saccharomyces cerevisiae* (Fig. S4). In both cases REAMP2.0 was associated with sedimenting membranes and could be purified in Cymal-5 as above. Purification from *R. sphaeroides* gave similar yields to *E. coli* while *S. cerevisiae* produced less protein, at 1.2 mg per g total membrane protein. REAMP2.0 thus appears to be broadly tolerated by diverse cells with different membrane compositions and biosynthetic machinery.

Irrespective of the recombinant source of REAMP2.0, size exclusion chromatography showed a single major peak indicative of a uniform product (Fig. 2b, Fig. S4c). In-line static light scattering (SEC-MALS) and native mass spectrometry confirmed that REAMP2.0 was a monodisperse monomer that was stable against aggregation (Fig. 2c,d). From SEC-MALS, the protein component of the protein-detergent complex (*PDC*) was 23 kDa^26^, and the protein-detergent complex comprised approximately 100 detergent molecules with δ = 2.04.

### Cells expressing REAMP2.0 accumulate zinc protoporphyrin IX

Cofactors such as heme can introduce functionality to de novo proteins^11,12,27–33^. To promote such cofactor binding in vivo, recombinant strains were supplemented with the heme precursor *δ*-aminolevulinic acid (ALA). Under these conditions cells expressing REAMP2.0, REAMP2.0^H^ and REAMP2.0^H/H^ produced a light red pigment that was identified by absorption spectroscopy, fluorimetry and mass spectrometry as zinc protoporphyrin IX (ZnPPIX). The data for REAMP2.0^H^ are taken as representative and are shown in Fig. 2e,f, Supplementary Table 2, and Fig. S6.

The accumulated ZnPPIX was fractionated mainly with cellular membranes, rather than with inclusion bodies or the cytoplasm. It was produced in addition to membrane heme (Fig. S7) at 0.4 μmol ZnPPIX per g total membrane protein, similar to the expression yield of REAMP2.0. The bioproduction of ZnPPIX was tunable with REAMP2.0^H^ expression (Fig. 2g, Fig. S8) and this was independent of any purification tag (Fig. S9). ZnPPIX was not observed in control strains overexpressing the endogenous diheme cytochrome CybB (Fig. 2h) and was only reduced by about one-third in strains supplemented with excess iron (Fig. 2h).

One possible explanation for these results is that REAMP2.0^H^ and ZnPPIX form a complex within the cell. To support this we confirmed that purified REAMP2.0^H^ bound to both demetallated and Zn-substituted PPIX in vitro (Fig. S10). However, affinity chromatography from pigmented cells only ever purified the REAMP2.0^H^ apoprotein, perhaps because cofactor binding by the protein is out-competed by partitioning into the large excess of empty detergent micelles. Purification with the detergent-free styrene maleic acid system^34^ was unsuccessful because of low membrane extraction efficiency. Further work will be required to elucidate the basis for ZnPPIX accumulation in REAMP2.0 strains.

### Structural characterization of REAMP2.0

**Figure 3:**
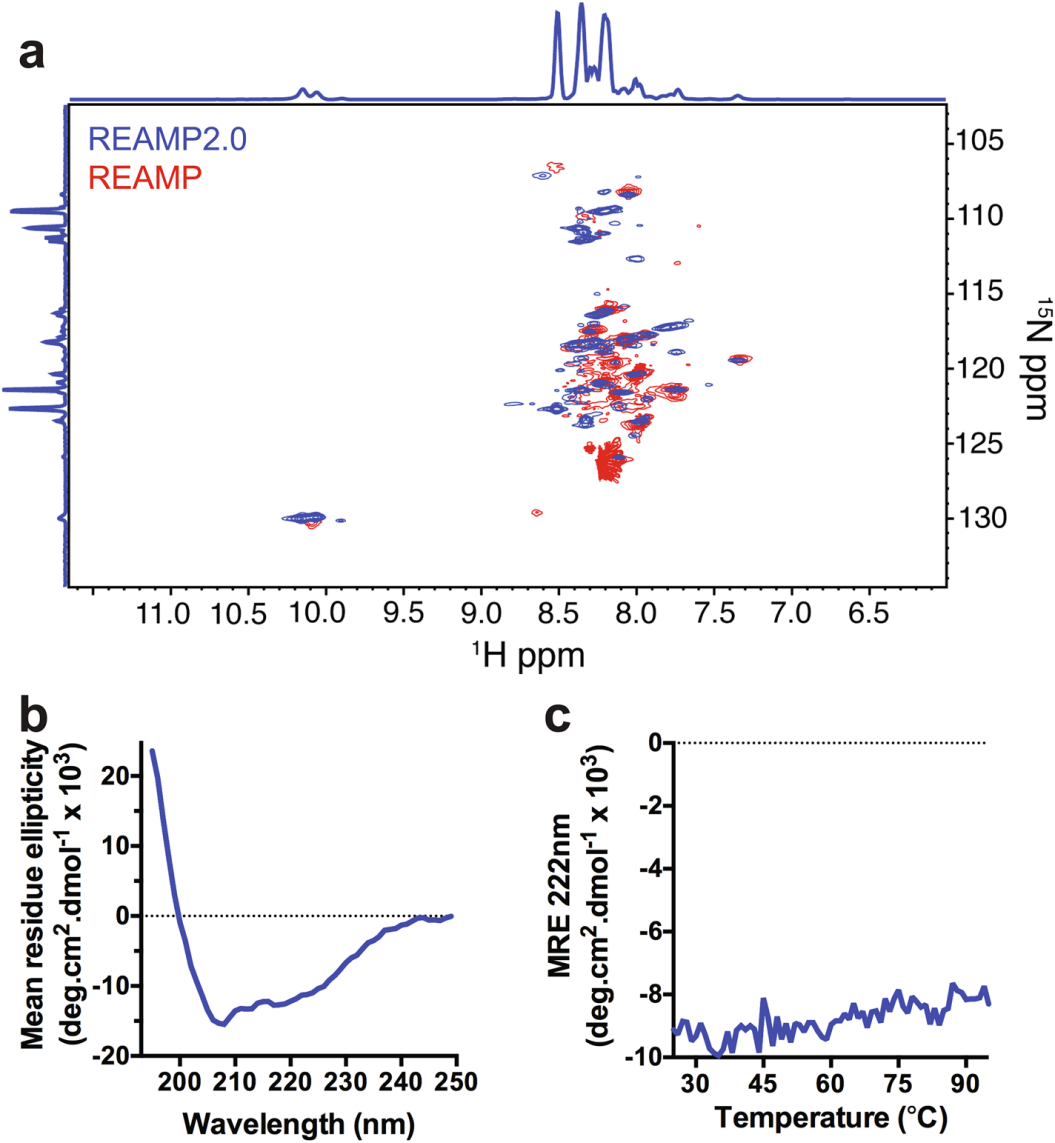
Low-resolution information on the structure of REAMP2.0. (**a**) The dispersity and resolution of ^1^H-^15^N TROSY-HSQC NMR spectra suggest that REAMP2.0 (*blue*) has improved packing relative to the parent REAMP design (*red*). A close-up of the glycine region is shown in Fig. S5. (**b**) UV-Circular Dichroism determines that REAMP2.0 is an α-helical protein in agreement with the design. (**c**) This helical secondary structure persists at high temperatures.

The successful purification of REAMP2.0 made it possible to explore the impact of the GAS_Left_ motif on protein folding and assembly. Structural analysis by ^1^H-^15^N TROSY-HSQC NMR yielded a spectrum with a greater number of sharp, resolvable resonances for REAMP2.0 compared with the original REAMP protein (Fig. 3a, Fig S5 and ref. 24). Improved spectral quality is usually an indicator of a reduction in protein conformational exchange and may indicate a shift to improved packing and folding in the second-generation design. For example, multiple resonances corresponding to the indole protons of tryptophan are now observable in REAMP2.0 versus a single broad correlation in REAMP. Moreover, the ^15^N chemical shift range around ~ 110 ppm is typically predominated by glycine amide resonances and numerous new resonances appear in this region of the ^1^H-^15^N HSQC of REAMP2.0 (enlarged in Fig. S5), implying a change in the folding environment around these small residues. The repetitive nature of the REAMP primary sequences precluded any further realistic attempt at chemical shift assignment. However, additional analysis by circular dichroism confirmed that REAMP2.0 was α-helical (Fig. 3b). The helicity was about 35%, consistent with design whereby 84 of 245 residues are expected to reside within the four transmembrane α-helices. This helicity persisted up to 95 °C, in common with the stability of transmembrane helices in other natural and designed membrane proteins.

### Heme binding as a probe of protein flexibility

**Figure 4:**
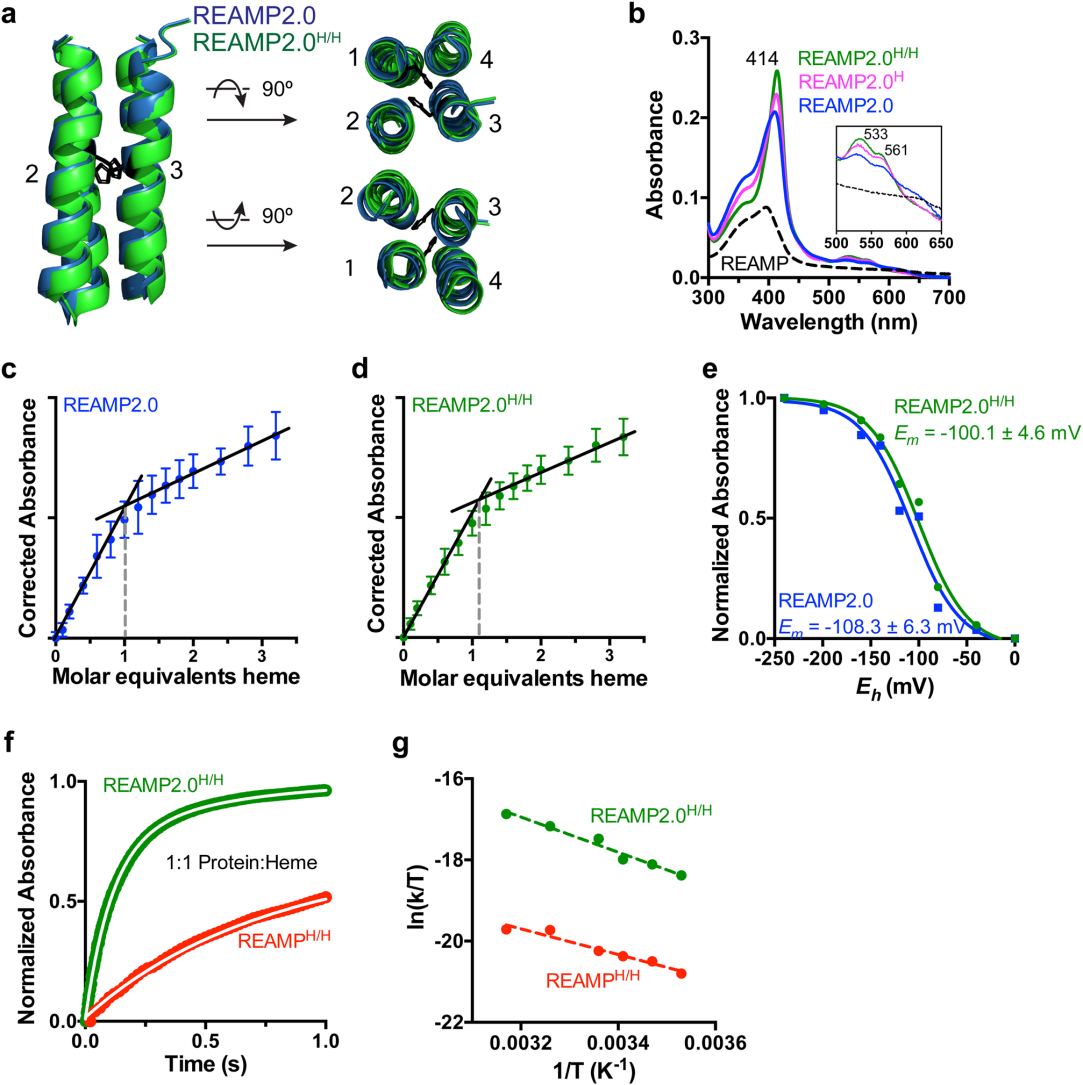
Heme binding by REAMP2.0 and histidine variants. (**a**) The protein core of REAMP2.0 can accommodate buried histidines in the mutant REAMP2.0^H/H^. (**b**) Absorption spectra at 1.5 μM of both protein and heme. Data for the prototype REAMP design are shown for comparison. (**c,d**) Equilibrium titrations consistent with tight binding, with a deflection point at 1 heme equivalent. A buffer background is subtracted from both curves. (**e**) Potentiometric redox titrations of heme complexes, fit to the one-electron Nernst equation. (**f**) Heme binding to REAMP2.0^H/H^ is much faster than to REAMP^H/H^. The overlaid white line is the fit to a biexponential function. (**g**) Eyring plots derived from initial rates are consistent with improved conformational definition in REAMP2.0^H/H^. See text for details.

We next sought to obtain further insight into the structure and dynamics of REAMP2.0 through cofactor binding in vitro^32^. Improvements in the packing order of a de novo protein should increase the enthalpic cost of cofactor binding (ΔH^‡^), because pre-organisation of the protein structure means that a greater number of intraprotein interactions must be broken for binding to occur. This ought to be compensated for by a decreased entropic penalty (ΔS^‡^), because there is less cofactor-induced ordering of the holoprotein relative to the apoprotein. We investigated the binding of a heme cofactor and so constructed protein variants with one or two buried histidines to allow axial coordination to the heme iron (Fig. 4a). These mutants were termed REAMP2.0^H^ and REAMP2.0^H/H^, respectively.

Purified REAMP2.0 was able to complex heme in vitro, in contrast to the original REAMP design (Fig. 4b). The ambient absorption spectrum was consistent with hydrophobic heme burial, with a Soret peak at ~ 414 nm and Q-bands at 533 nm and 561 nm. Both REAMP2.0^H^ and REAMP2.0^H/H^ showed slight sharpening of the Soret and Q-bands consistent with histidine coordination to the heme iron (Fig. 4b). Heme titrations produced tight binding curves with a deflection point at one equivalent of heme per protein (Fig. 4c,d). Binding of a single heme was also inferred from the single midpoint redox potential (*E*_*m*_) observed at around − 100 mV for all three constructs (Fig. 4e, Fig. S11, Supplementary Table 3). The potentials measured here are very similar to those previously obtained for REAMP bis-His variants that can complex heme^24^, implying that the immediate heme environment is consistent between the two designs.

The binding of heme by REAMP2.0^H/H^ was an order of magnitude faster than to REAMP^H/H^, with the data fitting to the sum of two exponential phases (Fig. 4f, Fig. S12). Following the work of Dutton^32^ we used initial rates to confirm that these data were consistent with a bimolecular second-order reaction (Fig. S13) and to determine the pseudothermodynamics of heme binding. Eyring plots (Fig. 4g) gave an apparent activation energy of binding, ΔG^‡^, for REAMP2.0^H/H^ of 26.1 kcal.mol^−1^, which was 1.6 kcal.mol^-1^ lower than for REAMP^H/H^. This change in ΔG^‡^ for REAMP2.0^H/H^ was associated with an increase in ΔH^‡^ and decrease in − TΔS^‡^, consistent with improved structural definition as discussed above. The values of ΔH^‡^, − TΔS^‡^ and ΔH^‡^ (Supplementary Table 4) were similar to those observed for water-soluble de novo proteins^32^.

### Structural rigidity can improve catalysis by heme

**Figure 5:**
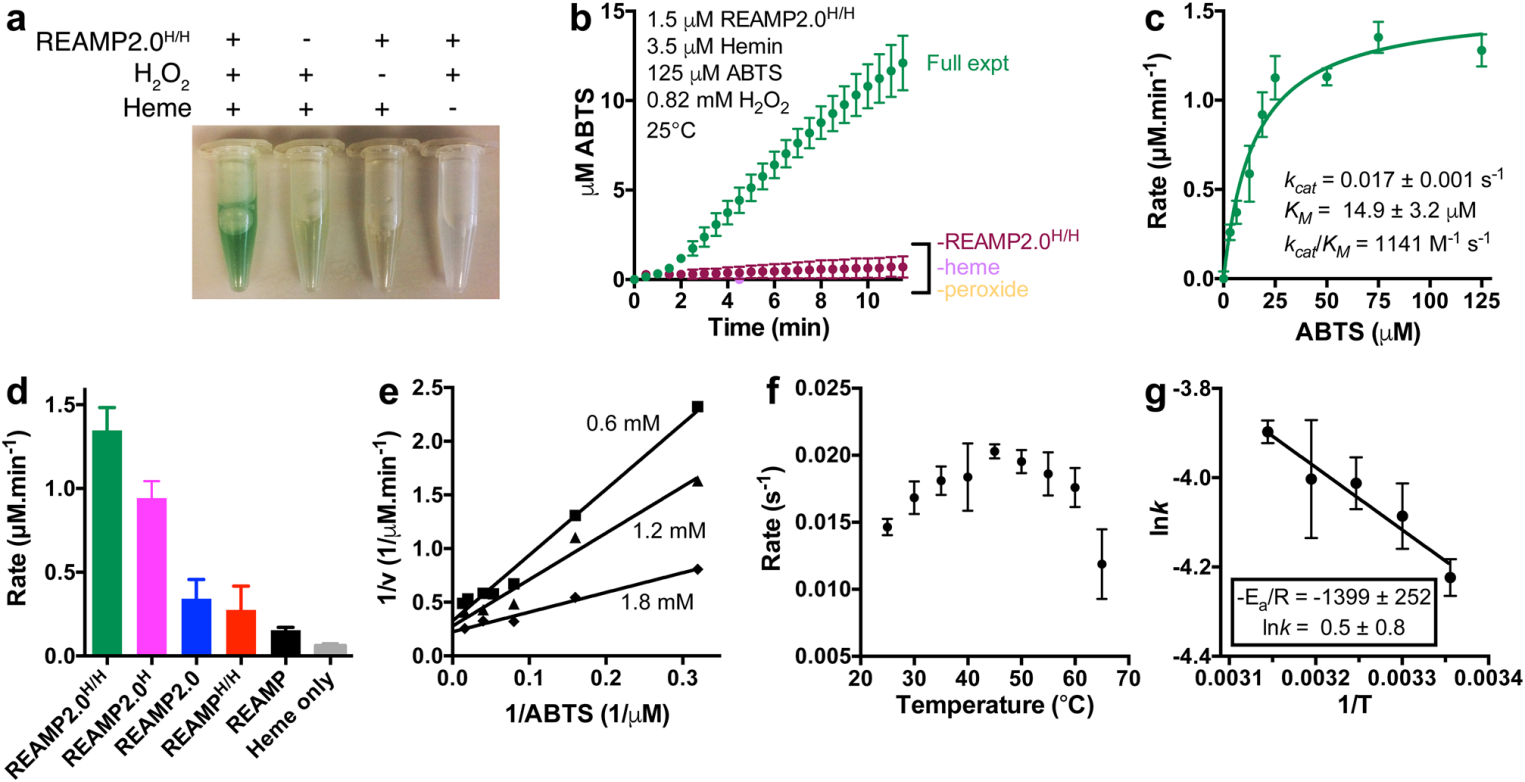
Peroxidase activity of REAMP2.0 heme complexes. (**a**,**b**) A REAMP2.0 hemoprotein catalyses ABTS oxidation by peroxide, generating a green product that is clearly resolved over controls omitting one of the reactants. (**c**) The reaction follows saturable kinetics. Solid line shows fitting to the Michaelis–Menten equation at pH 7.4, 0.82 mM H_2_O_2_. (**d**) The bis-histidine variant of REAMP2.0 has the highest activity among the REAMPs tested. (**e**) Diagnostic double-reciprocal plot of REAMP2.0^H/H^ at different peroxide concentrations as shown. (**f**) Optimum assay temperature is 42 °C. (**g**) Arrhenius plot at non-denaturing temperatures. All panels except (**e**) show mean ± SD of three independent repeats.

We previously found that REAMP^H/H^ was marginally active as a heme peroxidase^24^, and so wondered if the dynamical changes observed in REAMP2.0 would affect this activity. Peroxidase assays confirmed that REAMP2.0 and its histidine variants were substantially more active than analogous REAMP complexes (Fig. 5a–d, Fig. S14). Activity was also markedly enhanced by the presence of axial histidines, being highest for REAMP2.0^H/H^. The classical peroxide substrate ABTS was used to determine Michaelis–Menten kinetics in excess peroxide (Fig. 5c). The catalytic efficiency *k*_*cat*_/*K*_*M*_ at pH 7.4 was 1,141 ± 254 M^−1^ s^−1^, with *k*_*cat*_ of 0.017 ± 0.001 s^−1^ and *K*_*M*_ of 14.9 ± 3.2 μM. The full reaction matrix could not be explored since increasing the buffer pH caused visible protein aggregation and very high peroxide concentrations appeared to degrade the heme. Data collected at different peroxide concentrations produced non-parallel double-reciprocal plots, indicative of a sequential Bi-Bi mechanism (Fig. 5e).

The optimum temperature for catalysis was 42 °C (Fig. 5f). REAMP2.0 does not unfold or aggregate at this temperature (Fig. 3c), but the structural integrity required for effective catalysis is apparently lost. An Arrhenius plot (Fig. 5g) determined an activation energy of 2.8 kcal.mol^−1^ and pre-exponential factor (*A*) of 1.6 s^−1^, the latter being many orders of magnitude lower than natural enzymes. This confirms that the catalytic activity of REAMPs is limited by a small number of productive substrate collisions at the cofactor site.

## Discussion

The de novo design of integral membrane proteins is an ‘acid test’ of how primary sequence dictates form and function in the complex membrane environment. Because natural membrane proteins have accumulated considerable complexity through evolutionary time, simple model systems have become a powerful way to explore the sequence basis of membrane protein trafficking, insertion, topology, and folding^35–37^. Here, we extend this reductionist approach to a minimal recombinant protein.

Our work demonstrates that REAMPs are amenable to recombinant expression (Fig. 2). Most de novo designs of membrane proteins have employed the chemical synthesis of short peptides that can assemble in model lipid bilayers^3,4,7,9–11^ and biological expression has received less attention^5,6,8,12^. The biosynthesis of de novo proteins is an enticing prospect since it could generate large constructs that are inaccessible to chemical synthesis, test the degree of novelty that can be tolerated by living systems and engage with the biochemistry of the living cell. However, membrane proteins have a somewhat deserved reputation for being intractable, with particular issues around expression yields, membrane localisation and successful assembly. Even if artificial sequences can be effectively produced, further hurdles lie in extracting the biosynthesised protein from the lipid bilayer in a state suitable for biophysical characterisation. A key question remains: how far can de novo sequences diverge from natural sequences before being rejected by the cell? REAMP2.0 is tolerated by sundry recombinant hosts, including purple bacteria and yeast, and can be purified from the membrane fractions of these cells in a well-folded, stable and monodisperse state. However, a substantial fraction of REAMP2.0 expressed in *E. coli* aggregates into cellular inclusion bodies that can no longer be recovered in gentle non-ionic surfactants. An interesting future design challenge will be to bias expression towards productive membrane localisation over the unproductive formation of intracellular aggregates.

Our results suggest that without any further optimisation, imprinting the GAS_Left_ packing code onto the REAMP sequence is sufficient to reduce the conformational heterogeneity of this protein. This in turn improves structurally-dependent properties such as cofactor binding and catalysis. In light of these results, we see the REAMPs as conceptually analogous to the pool of structurally plastic and functionally promiscuous primordial sequences that were the likely ancestors of modern proteins^38^. The relative simplicity of the REAMPs means they can be used to explore how innovations in such ancestral sequences may have supported the acquisition of particular structures or activities. There are relatively few packing modes found in natural modern membrane proteins^14,39^, and evolutionary time has not been sufficient to sample all possible sequence combinations^40^. REAMPs could potentially be used to discover helix packing interfaces that have not arisen through natural selection, and so to generate novel protein architectures.

We also report the surprising finding that strains of *E. coli* expressing REAMP2.0 accumulate ZnPPIX when supplemented with the porphyrin precursor ALA. ZnPPIX does occur naturally in biological systems, but is rather rare^41^. The Zn metal center is capable of light-activated electron transfer, and so could be the basis for synthetic pigment proteins capable of light harvesting, metal sensing and photocatalysis. The data here must be considered provisional, and might simply arise from lower-order hydrophobic protein aggregates that can sequester the cofactor. Regardless of the precise mechanism of interaction, we speculate that REAMP2.0 can act as a sink for the co-ordination of demetallated or ‘free base’ porphyrin within the cell, and that this complex is then non-enzymatically metallated with Zn. It is interesting here to note the very low bioavailability of Zn in *E. coli*—estimated at less than one free atom per cell^42^. It thus appears that REAMP2.0 can either outcompete or bypass cellular zinc stores.

Collectively, our results show that a rational, knowledge-based approach can improve the structural uniqueness and function of a genetically-encoded artificial membrane protein. This provides further empirical support for incorporating natural sequence patterns, such as the GAS_Left_ motif, into de novo designs. While such packing motifs by themselves cannot tell the full story of membrane protein folding^43^, our work confirms the relevance of considering these sequence codes as part of the design process.

## Materials and methods

### Computational modeling

Preliminary models of REAMP2.0 were constructed as follows. The four alpha-helices of a synthetic antiparallel homotetramer (PDB 3R4A) were converted to polyalanine and helices B and C realigned to avoid any orientation bias. Loops were introduced using the Chimera interface to Modeller. The sequence was then mutated to REAMP2.0 and SCWRL4 used to pack the sidechains. This entire initial model (including loops and C-terminal tags) was used for further packing simulations with RosettaMP^44^ using the score function mpframework_smooth_fa_2012.wts with 10,000 decoys. The resulting models were rescored considering only the transmembrane domain as specified by the Rosetta span file. RMSD was calculated relative to the lowest-scoring decoy. Mutations were introduced with mp_mutate_relax^45^. The models and protocols are provided as additional supplementary data.

### Protein expression

REAMP2.0 was obtained from ATUM, Inc as a synthetic gene optimized for bacterial expression. For recombinant production in *E. coli* this gene was cloned into pET28 by cohesive end ligation after restriction digest with NcoI and XhoI. Either decahistidine or triple *Strep*II-tag sequences were placed at the C-terminus for affinity purification. Culturing was performed as previously^24^ in the commercial strain BL21-AI (Invitrogen) and protein expression was induced at A600 = 0.9 with 0.1% arabinose and 0.1 mM IPTG for 2 h.

### Protein purification

Protein purification from *E. coli* was as previously described^24^ with no modifications. The process is outlined in Fig. 2a. Briefly, cells were lysed under pressure using a cell disrupter (Constant Systems) at 25 KPSI. After the lysate was clarified by centrifugation at 10,000 *g*, membranes were isolated at 170,000 *g* and resuspended to 5 mg/ml in Buffer A (50 mM Tris buffer, pH 8.0, 150 mM NaCl, 5% glycerol) prior to solubilisation in 2.4% Cymal-5. Soluble membranes were applied to a 1 ml *Strep*-Tactin or His-Trap column equilibrated in Buffer A plus 0.24% Cymal-5, washed in at least 20 column volumes of the same (including 75 mM imidazole for the His-tagged protein), and eluted with 2.5 mM *d*-desthiobiotin or 0.5 M imidazole. A similar method was used for yeast purification except that cell disruption was at 35 KPSI. Membranes from *Rhodobacter sphaeroides* were fractionated on a 60:40 step sucrose gradient before proceeding with detergent solubilization. The theoretical molecular weight of the REAMP2.0 *Strep*II-tag construct is 22,945 with an extinction coefficient of 60,500 M^−1^.cm^−1^.

### Protein analysis

SDS-PAGE, size exclusion chromatography and circular dichroism were all performed as previously described^24^. Analysis by static light scattering (SEC-MALS) used the three-detector method described by Slotboom^26^, with a calculated refractive index increment of 0.184 ml/g for REAMP2.0 and 0.152 ml/g for Cymal-5. Heteronuclear 2D NMR was performed after ^15^N labelling in minimal media^24,46,47^. Samples were at 9 mg/ml in Buffer A plus 0.24% Cymal-5 with 10% D_2_O. Data were collected on a Bruker Avance-III-700 equipped with a 1.7 mm TXI Z-gradient probe at 313 K. The ^1^H-^15^N BEST-TROSY spectra were acquired with a spectral width of 14 ppm in ^1^H and 33 ppm in ^15^N with a relaxation delay of 0.2 s using the b_trosyf3gpph.2 pulse program.

### Cofactor binding

Purified apoproteins were diluted into Buffer A plus 0.012% Cymal-5. Heme (as hemin) or zinc protoporphyrin IX were introduced and samples incubated at 25 °C for equilibrium measurements. Redox potentiometry was performed in the presence of mediators as before^24^. For kinetic analysis, heme and protein were mixed at 1 μM each reactant in a stopped-flow instrument in absorption mode. The kinetics signal was transformed to the concentration of bound heme by reference to a standard curve. The entirety of the dataset was fit to the sum of two exponential functions and early timepoints fit to a linear function. Kinetic data at different temperatures was fit to the linear form of the Eyring equation (Eq. 1), assuming a transmission coefficient of 1:

1 $${\ln}\left( {k/{\text{T}}} \right) \, = \, - \Delta {\text{H}}^{\ddag } /{\text{RT }} + {\ln}\left( {k_{B} /h} \right) \, + \Delta{\text{S}}^{\ddag } /{\text{R}}$$

All data fitting was carried out in GraphPad Prism.

### Peroxidase assays

Reactions were generally 1.5 μM protein and 3 μM hemin in 400 μl Buffer A plus 0.012% Cymal-5 at 25 °C. The substrates 2,2′-Azinobis[3-ethylbenzothiazoline-6-sulfonic acid] (ABTS) and hydrogen peroxide were introduced at varying concentrations as required.

### Extraction of cellular porphyrins

To promote porphyrin production LB media was supplemented with 0.3 μM δ-aminolevulinic acid at the point of protein induction. Where required, ferrous iron was supplied as 0.1 mM ammonium iron sulphate. Cell membranes from 1L culture were isolated and adjusted to 5 mg/ml total protein. Aliquots of this membrane suspension were pelleted at 13,000 g, the supernatant was discarded, and the pellet resuspended by extensive pipetting in 80/20/1 (v/v/v) Ethanol/DMSO/acetic acid^48^. After a short time the sample was centrifuged at 13,000 g and the organic extract in the supernatant removed for spectroscopy or mass spectrometry. Alternatively, the cell pellet from 50 ml of induced culture was resuspended in 1 ml ‘BugBuster’ reagent (Merck Millipore) and incubated for 1 h. This sample was centrifuged at 13,000 *g* for 10 min and the supernatant taken for spectroscopy and immunoblotting.

## Supplementary information

Supplementary file1

Supplementary file2

## References

[1] Ghirlanda G (2009). Design of membrane proteins: Toward functional systems. Curr. Opin. Chem. Biol..

[2] Barth P, Senes A (2016). Towards high-resolution computational design of the structure and function of helical membrane proteins. Nat. Struct. Mol. Biol..

[3] Kennedy SJ, Roeske RW, Freeman AR, Watanabe AM, Besche HR (1977). Synthetic peptides form ion channels in artificial lipid bilayer membranes. Science.

[4] Lear JD, Wasserman ZR, DeGrado WF (1988). Synthetic amphiphilic peptide models for protein ion channels. Science.

[5] Heim EN (2015). Biologically active LIL proteins built with minimal chemical diversity. PNAS.

[6] Whitley P, Nilsson I, von Heijne G (1994). de novo design of integral membrane proteins. Nat. Struct. Biol..

[7] Mravic M (2019). Packing of apolar side chains enables accurate design of highly stable membrane proteins. Science.

[8] Lu P (2018). Accurate computational design of multipass transmembrane proteins. Science.

[9] Mahendran KR (2017). A monodisperse transmembrane alpha-helical peptide barrel. Nat. Chem..

[10] Joh NH (2014). De novo design of a transmembrane Zn^2+^-transporting four-helix bundle. Science.

[11] Korendovych IV (2010). *De novo* design and molecular assembly of a transmembrane diporphyrin-binding protein complex. J. Am. Chem. Soc..

[12] Goparaju G (2016). First principles design of a core bioenergetic transmembrane electron-transfer protein. Biochim. Biophys. Acta Bioenerg..

[13] Luckey M (2011). Membrane Structural Biology: With Biochemical and Biophysical Foundations.

[14] Walters R, DeGrado WF (2006). Helix-packing motifs in membrane proteins. PNAS.

[15] Senes A, Gerstein M, Engelman DM (2000). Statistical analysis of amino acid patterns in transmembrane helices: The GxxxG motif occurs frequently and in association with beta-branched residues at neighbouring positions. J. Mol. Biol..

[16] Russ WP, Engelman DM (2000). The GxxxG motif: A framework for transmembrane helix-helix association. J. Mol. Biol..

[17] Teese MG, Langosch D (2015). Role of GxxxG motifs in transmembrane domain interactions. Biochemistry.

[18] Mueller BK, Subramaniam S, Senes A (2014). A frequent, GxxxG-mediated, transmembrane association motif is optimized for the formation of interhelical Calpha-H hydrogen bonds. PNAS.

[19] Senes A, Ubarretxena-Belandia I, Engelman DM (2001). The Calpha-H…O hydrogen bond: A determinant of stability and specificity in transmembrane helix interactions. PNAS.

[20] Kim S (2005). Transmembrane glycine zippers: Physiological and pathological roles in membrane proteins. PNAS.

[21] Tatko CD, Nanda V, Lear JD, DeGrado WF (2006). Polar networks control oligomeric assembly in membranes. JACS.

[22] Choma C, Gratkowski H, Lear JD, DeGrado WF (2000). Asparagine-mediated self-association of a model transmembrane helix. Nat. Struct. Biol..

[23] Anderson SM, Mueller BK, Lange EJ, Senes A (2017). Combination of Calpha-H hydrogen bonds and van der Waals packing modulates the stability of GxxxG-mediated dimers in membranes. J. Am. Chem. Soc..

[24] Lalaurie CJ (2018). The *de novo* design of a biocompatible and functional integral membrane protein using minimal sequence complexity. Sci. Rep..

[25] Sparks KA (2014). Comparisons of interfacial Phe, Tyr, and Trp residues as determinants of orientation and dynamics for GWALP transmembrane peptides. Biochemistry.

[26] Slotboom D, Duurkens R, Olieman K, Erkens G (2008). Static light scattering to characterize membrane proteins in detergent solution. Methods.

[27] Farid TA (2013). Elementary tetrahelical protein design for diverse oxidoreductase functions. Nat. Chem. Biol..

[28] Koder RL (2009). Design and engineering of an O_2_ transport protein. Nature.

[29] Choma CT (1994). Design of a heme-binding four-helix bundle. JACS.

[30] Discher BM (2005). Design of amphiphillic protein maquettes: controlling assembly, membrane insertion, and cofactor interactions. Biochemistry.

[31] Huang SS, Koder RL, Lewis M, Wand AJ, Dutton PL (2004). The HP-1 maquette: from an apoprotein structure to a structured hemoprotein designed to promote redox-coupled proton exchange. PNAS.

[32] Solomon LA, Kodali G, Moser CC, Dutton PL (2014). Engineering the assembly of heme cofactors in man-made proteins. JACS.

[33] Robertson DE (1994). Design and synthesis of multi-haem proteins. Nature.

[34] Lee SC (2016). A method for detergent-free isolation of membrane proteins in their local lipid environment. Nat. Protoc..

[35] Killian JA, Nyholm TK (2006). Peptides in lipid bilayers: the power of simple models. Curr. Opin. Struct. Biol..

[36] Rath A, Tulumello D, Deber CM (2009). Peptide models of membrane protein folding. Biochemistry.

[37] Cymer F, von Heijne G, White SH (2015). Mechanisms of integral membrane protein insertion and folding. J. Mol. Biol..

[38] James LC, Tawfik DS (2003). Conformational diversity and protein evolution—a 60-year-old hypothesis revisited. Trends Biochem. Sci..

[39] Feng X, Barth P (2016). A topological and conformational stability alphabet for multipass membrane proteins. Nat. Chem. Biol..

[40] Woolfson DN (2015). *De novo* protein design: How do we expand into the universe of possible protein structures?. Curr. Opin. Struct. Biol..

[41] Labbé RF, Vreman HJ, Stevenson DK (1999). Zinc protoporphyrin: a metabolite with a mission. Clin. Chem..

[42] Outten CE, O’Halloran TV (2001). Femtomolar sensitivity of metalloregulatory proteins controlling zinc homeostasis. Science.

[43] Li E, Wimley WC, Hristova K (2012). Transmembrane helix dimerisation: beyond the search for sequence motifs. Biochim. Biophys. Acta Biomembr..

[44] Alford R (2015). An integrated framework advancing membrane protein modeling and design. PLoS Comput. Biol..

[45] Koehler Leman J, Mueller BK, Gray JJ (2018). Expanding the toolkit for membrane protein modeling in Rosetta. Bioinformatics.

[46] Delaglio F (1995). NMRPipe: a multidimensional spectral processing system based on UNIX pipes. J. Biomol. NMR.

[47] Marley J, Lu M, Bracken C (2001). A method for efficient isotopic labeling of recombinant protein. J. Biomol. NMR.

[48] Létoffé S, Heuck G, Delepelaire P, Lange N, Wandersman C (2009). Bacteria capture iron from heme by keeping tetrapyrrol skeleton intact. PNAS.

