# Supplementary material for: Small-residue packing motifs modulate the structure and function of a minimal de novo membrane protein
Source: Sci Rep. 2020 Sep 16;10:15203. doi: 10.1038/s41598-020-71585-8 (PMC7495484; doi:10.1038/s41598-020-71585-8)
Supplement: Supplementary file 1 — Supplementary file1 [file 41598_2020_71585_MOESM1_ESM.docx]

**SUPPLEMENTARY INFORMATION**

**Small-residue packing motifs modulate the structure and function of a minimal *de novo* membrane protein**

Paul Curnow^1,3*^, Benjamin J Hardy^1,3^, Virginie Dufour^1,3^, Christopher J Arthur^2^, Richard Stenner^1^, Lorna R Hodgson^1,3^, Paul Verkade^1,3^, Chris Williams^2,3^, Deborah K Shoemark^1,3^, Richard B Sessions^1,3^, Matthew P Crump^2,3^ Michael R Jones^1,3^ and JL Ross Anderson^1,3^

**Affiliations**: ^1^School of Biochemistry and ^2^School of Chemistry, University of Bristol, UK. ^3^BrisSynBio, Life Sciences Building, Tyndall Avenue, Bristol, BS8 1TQ, UK.

*Corresponding author. Email: p.curnow@bristol.ac.uk

Supplementary Table 1: Bioinformatic analyses predict that 4TM2.0 is a multipass membrane protein comprising four transmembrane α-helices with N_in_/C_in_ topology. TMPred was accessed via <http://embnet.vital-it.ch/software/TMPRED_form.html>; TMHMM, <http://www.cbs.dtu.dk/services/TMHMM/>; HMMTOP, <http://www.enzim.hu/hmmtop/>; Philius, via the TOPCONS^1^ server <http://topcons.net>; SCAMPI2, <http://scampi.bioinfo.se>.

| **Program** | **N** | **H1** | **H2** | **H3** | **H4** | **C** |  |
| --- | --- | --- | --- | --- | --- | --- | --- |
| TMHMM^2^ | in | 2-24 | 49-71 | 99-121 | 147-169 | in |  |
| TMPred | in | 3-21 | 53-70 | 99-117 | 149-166 | in |  |
| HMMTOP^3^ | No TM helices predicted | | | | | | |
| SCAMPI2^4^ | in | 2-22 | 49-70 | 98-118 | 146-166 | in |  |
| Philius^5^ | in | 1-28^‡^ | 51-71 | 98-118 | 146-166 | in |  |

^‡^As signal peptide.

Supplementary Table 2: Peak assignments from absorption and fluorescence spectroscopy of porphyrins and cell extracts. Values in parenthesis are normalized signal intensities at the peak maxima shown.

|  |  | **Absorption** | | | **Fluorescence** | | | |
| --- | --- | --- | --- | --- | --- | --- | --- | --- |
| **Sample** | **Condition** | **Soret** | **α** | **β** | **Emission^a^** | | **Excitation^b^** | |
| **REAMP2.0^H^ membranes** | FC-12 extract^c^ | 420 (1) | 548 (0.18) | 587 (0.18) | nd | nd | nd | nd |
|  | Ethanol/DMSO extract^d^ | 418 (1) | 547 (0.08) | 584 (0.08) | 589 (1) | 645 (0.33) | 547 (0.80) | 585 (1) |
|  | Pyridine Hemochrome^e^ | 422 (1) | 550 (0.09) | 586 (0.08) | 592 (1) | 646 (0.40) | 549 (0.86) | 586 (1) |
| **Cell extract** | BugBuster | nd | nd | nd | 590 (1) | 642 (0.4) | nd | nd |
| **Commercial ZnPPIX** | Ethanol/DMSO extract | 419 (1) | 547 (0.07) | 584 (0.07) | 590 (1) | 645 (0.34) | 548 (0.84) | 584 (1) |
|  | Pyridine Hemochrome | 423 (1) | 550 (0.15) | 587 (0.14) | 592 (1) | 645 (0.38) | 551 (1.00) | 587 (1) |
|  | Aqueous buffer | 409 (1) | 557 (0.12) | 590 (0.15) | 587 (1) | 641 (0.45) | 544 (0.92) | 580 (1) |
|  | Cymal5 micelles | 421 (1) | 548 (0.07) | 585 (0.08) | 590 (1) | 643 (0.33) | 547 (0.79) | 584 (1) |
|  | REAMP2.0^H^ apoprotein^f^ | 425 (1) | 552 (0.11) | 589 (0.12) | 595 (1) | 650 (0.45) | 551 (0.96) | 587 (1) |

^a^Excitation at 420 or 550 nm. ^b^Emission at 640 nm. ^c^FC-12, surfactant fos-choline-12. ^d^Membrane extract in 80:20:1 v/v Ethanol:DMSO:acetic acid. ^e^Hemochrome formed in the organic solvent extract. ^f^Spectra obtained after introducing ZnPPIX to purified REAMP2.0^H^ apoprotein.

Supplementary Table 3. Redox potentials of constructs tested previously and in the current study.

| **Sample** | ***Em* (mV)** | **Reference** |
| --- | --- | --- |
| Micellized Heme | *ca.* -130 | ^6^ |
| REAMP | -133 ± 3 | ^6^ |
| REAMP^H/H^ | -101 ± 5 | ^6^ |
| REAMP2.0 | -108 ± 6 | This work |
| REAMP2.0^H^ | -104 ± 4 | This work |
| REAMP2.0^H/H^ | -100 ± 5 | This work |

Supplementary Table 4. Energetics of heme binding to REAMP^H/H^ and REAMP2.0^H/H^ at 25°C.

| Protein | Initial rate (μM^-1^.s^-1^) | ΔH^‡^ (kcal.mol^-1^) | -TΔS^‡^ (kcal.mol^-1^) | ΔG^‡^ (kcal.mol^-1^) |
| --- | --- | --- | --- | --- |
| REAMP^H/H^ | 0.5 ± 0.03 | 6.3 ± 0.7 | 19.8 | 26.1 |
| REAMP2.0^H/H^ | 7.6 ± 0.2 | 8.6 ± 0.7 | 15.9 | 24.5 |

**SUPPLEMENTARY FIGURE S1**

>REAMP2.0_gene

ATGGTCTGGGCGTTGTTATCTGGTTTAGGTGCACTGTTGTTGTCGTTGCTGGGCTTGTTGTGGGCAAGCTCTAGCAGCGGTGGCGAAGAGGGTGGCAGCTCCTCTTCTAGCTCTAGCTCTGGTGGCGAGGAGGGCGGCAGCTCCAGCTCCTGGGCTCTGCTGAGCGGCCTGGGCGCCCTGCTGCTGAGCCTGCTGGGTTTGCTGTGGGCGAGCAGCAGCTCCGGTGGCAAGAAAGGTGGCTCCTCCAGCTCTAGCAGCAGCAGCGGCGGCAAAAAGGGCGGTTCAAGCTCCAGCTGGGCGCTTCTGAGCGGTCTGGGTGCGCTGCTGTTGAGCTTGCTGGGTCTGTTGTGGGCGTCTAGCTCCAGCGGTGGTGAAGAGGGCGGTAGCTCGTCCAGCTCCAGCAGCAGCGGTGGCGAAGAGGGTGGTTCCAGCTCGTCCTGGGCGCTGCTGTCGGGCCTGGGTGCTCTGCTGCTGTCTCTGCTGGGTCTGCTGTGGGCCAGCAGCGGTAAGCCGATTCCGAACCCGCTGTTAGGTCTGGACAGCACCAGCCTCGAGGGTGGTGGGTCTGGTGGTGGGAGCGGTGGAGGCAGCTGGTCGCATCCGCAGTTTGAGAAGGGCGGCGGATCAGGCGGCGGATCCGGCGGTGGCTCGTGGTCCCATCCGCAATTCGAGAAGGGTGGCGGCAGTGGTGGCGGCTCTGGCGGTGGGTCGTGGAGCCACCCACAGTTCGAAAAGTGA

>REAMP2.0_translated

MVWALLSGLGALLLSLLGLLWASSSSGGEEGGSSSSSSSSGGEEGGSSSSWALLSGLGALLLSLLGLLWASSSSGGKKGGSSSSSSSSGGKKGGSSSSWALLSGLGALLLSLLGLLWASSSSGGEEGGSSSSSSSSGGEEGGSSSSWALLSGLGALLLSLLGLLWASSGKPIPNPLLGLDSTSLEGGGSGGGSGGGSWSHPQFEKGGGSGGGSGGGSWSHPQFEKGGGSGGGSGGGSWSHPQFEK-

>REAMP2.0_GFP

ATGGTCTGGGCGTTGTTATCTGGTTTAGGTGCACTGTTGTTGTCGTTGCTGGGCTTGTTGTGGGCAAGCTCTAGCAGCGGTGGCGAAGAGGGTGGCAGCTCCTCTTCTAGCTCTAGCTCTGGTGGCGAGGAGGGCGGCAGCTCCAGCTCCTGGGCTCTGCTGAGCGGCCTGGGCGCCCTGCTGCTGAGCCTGCTGGGTTTGCTGTGGGCGAGCAGCAGCTCCGGTGGCAAGAAAGGTGGCTCCTCCAGCTCTAGCAGCAGCAGCGGCGGCAAAAAGGGCGGTTCAAGCTCCAGCTGGGCGCTTCTGAGCGGTCTGGGTGCGCTGCTGTTGAGCTTGCTGGGTCTGTTGTGGGCGTCTAGCTCCAGCGGTGGTGAAGAGGGCGGTAGCTCGTCCAGCTCCAGCAGCAGCGGTGGCGAAGAGGGTGGTTCCAGCTCGTCCTGGGCGCTGCTGTCGGGCCTGGGTGCTCTGCTGCTGTCTCTGCTGGGTCTGCTGTGGGCCAGCAGCGGTAAGCCGATTCCGAACCCGCTGTTAGGTCTGGACAGCACCAGCCTCGAGGGTGGTGGGTCTGGTGGTGGGAGCGGTGGAGGCAGCTGGTCGCATCCGCAGTTTGAGAAGGGCGGCGGATCAGGCGGCGGATCCCTGGTGCCGCGCGGCAGCAGTAAAGGAGAAGAACTTTTCACTGGAGTTGTCCCAATTCTTGTTGAATTAGATGGTGATGTTAATGGGCACAAATTTTCTGTCCGTGGAGAGGGTGAAGGTGATGCTACAAACGGAAAACTCACCCTTAAATTTATTTGCACTACTGGAAAACTACCTGTTCCGTGGCCAACACTTGTCACTACTCTGACCTATGGTGTTCAATGCTTTTCCCGTTATCCGGATCACATGAAACGGCATGACTTTTTCAAGAGTGCCATGCCCGAAGGTTATGTACAGGAACGCACTATATCTTTCAAAGATGACGGGACCTACAAGACGCGTGCTGAAGTCAAGTTTGAAGGTGATACCCTTGTTAATCGTATCGAGTTAAAGGGTATTGATTTTAAAGAAGATGGAAACATTCTTGGACACAAACTGGAGTACAACTTTAACTCACACAATGTATACATCACGGCAGACAAACAAAAGAATGGAATCAAAGCTAACTTCAAAATTCGCCACAACGTTGAAGATGGTTCCGTTCAACTAGCAGACCATTATCAACAAAATACTCCAATTGGCGATGGCCCTGTCCTTTTACCAGACAACCATTACCTGTCGACACAATCTGTCCTTTCGAAAGATCCCAACGAAAAGCGTGACCACATGGTCCTTCTTGAGTTTGTAACTGCTGCTGGGATTACACATGGCATGGATGAGCTCTACAAATGA

>REAMP2.0_GFP_translated

MVWALLSGLGALLLSLLGLLWASSSSGGEEGGSSSSSSSSGGEEGGSSSSWALLSGLGALLLSLLGLLWASSSSGGKKGGSSSSSSSSGGKKGGSSSSWALLSGLGALLLSLLGLLWASSSSGGEEGGSSSSSSSSGGEEGGSSSSWALLSGLGALLLSLLGLLWASSGKPIPNPLLGLDSTSLEGGGSGGGSGGGSWSHPQFEKGGGSGGGSLVPRGSSKGEELFTGVVPILVELDGDVNGHKFSVRGEGEGDATNGKLTLKFICTTGKLPVPWPTLVTTLTYGVQCFSRYPDHMKRHDFFKSAMPEGYVQERTISFKDDGTYKTRAEVKFEGDTLVNRIELKGIDFKEDGNILGHKLEYNFNSHNVYITADKQKNGIKANFKIRHNVEDGSVQLADHYQQNTPIGDGPVLLPDNHYLSTQSVLSKDPNEKRDHMVLLEFVTAAGITHGMDELYK

Figure S1. Synthetic gene sequences used in this work, and the corresponding gene products.

**SUPPLEMENTARY FIGURE S2**

**
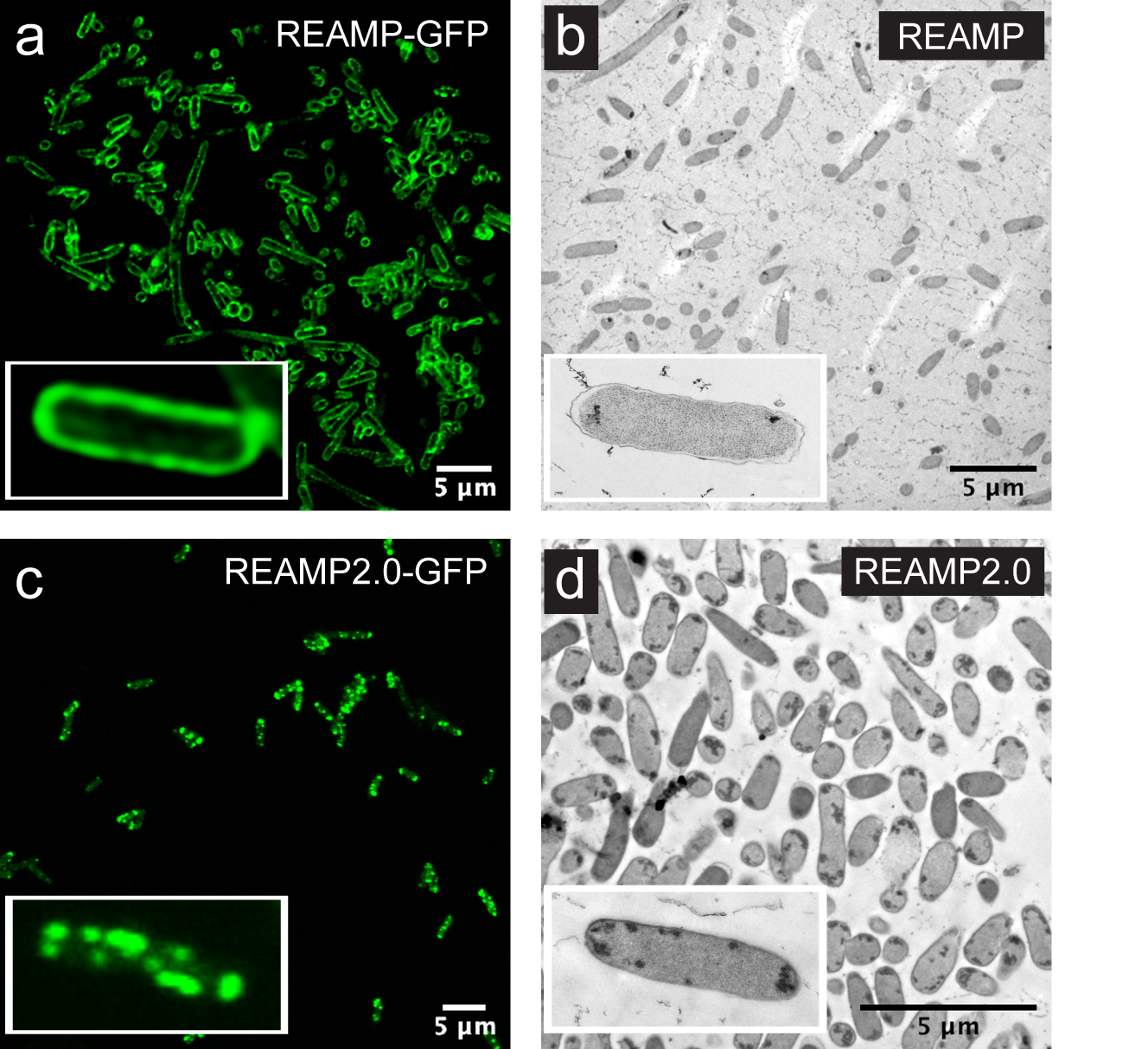
**

Figure S2. Cellular expression of the *de novo* membrane proteins REAMP and REAMP2.0 in *E. coli*. (**a**) Confocal light microscopy confirms that the parent design, REAMP, is efficiently localised to cell membranes. (**b**) Electron microscopy shows that minor electron-dense aggregates are seen in the cytoplasm of some REAMP cells. (**c, d**) In contrast, the second-generation design REAMP2.0 accumulates mainly as cytoplasmic inclusions.

**SUPPLEMENTARY FIGURE S3**


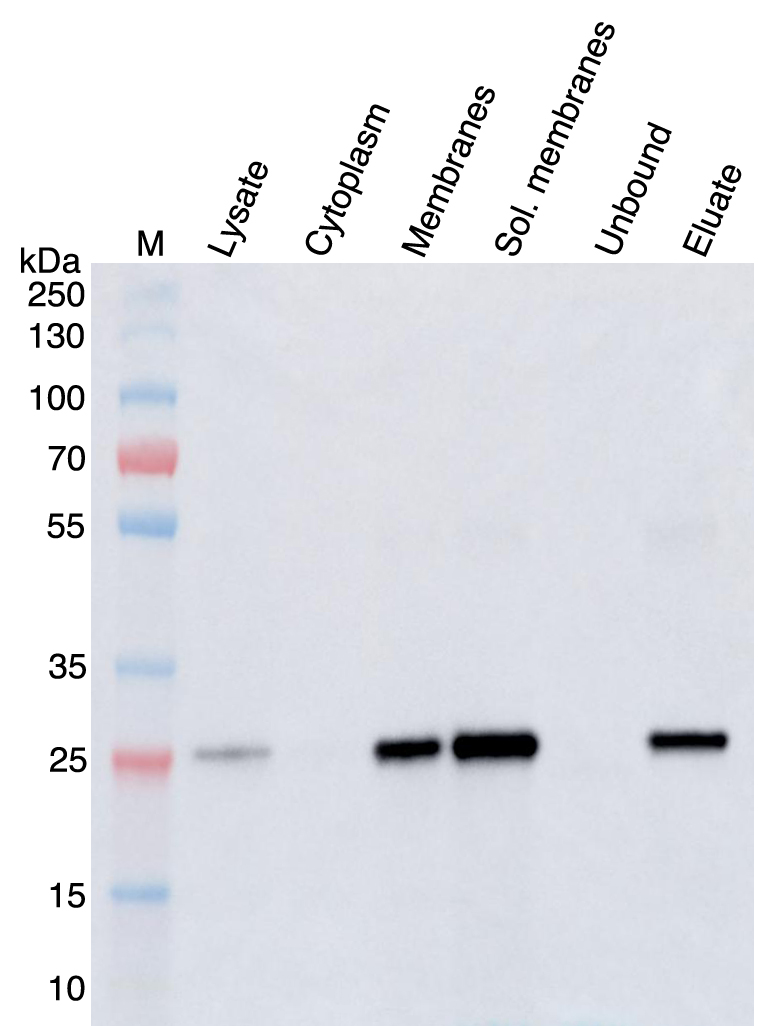


Figure S3. Western blotting with anti-V5 tracks the fate of REAMP2.0 during purification. A cropped version of this gel is shown in Figure 2 of the main text. All lanes are loaded with 10 micrograms of total protein, as determined by Lowry assay, apart from the *Eluate* which is 0.25 micrograms total protein.

**SUPPLEMENTARY FIGURE S4**


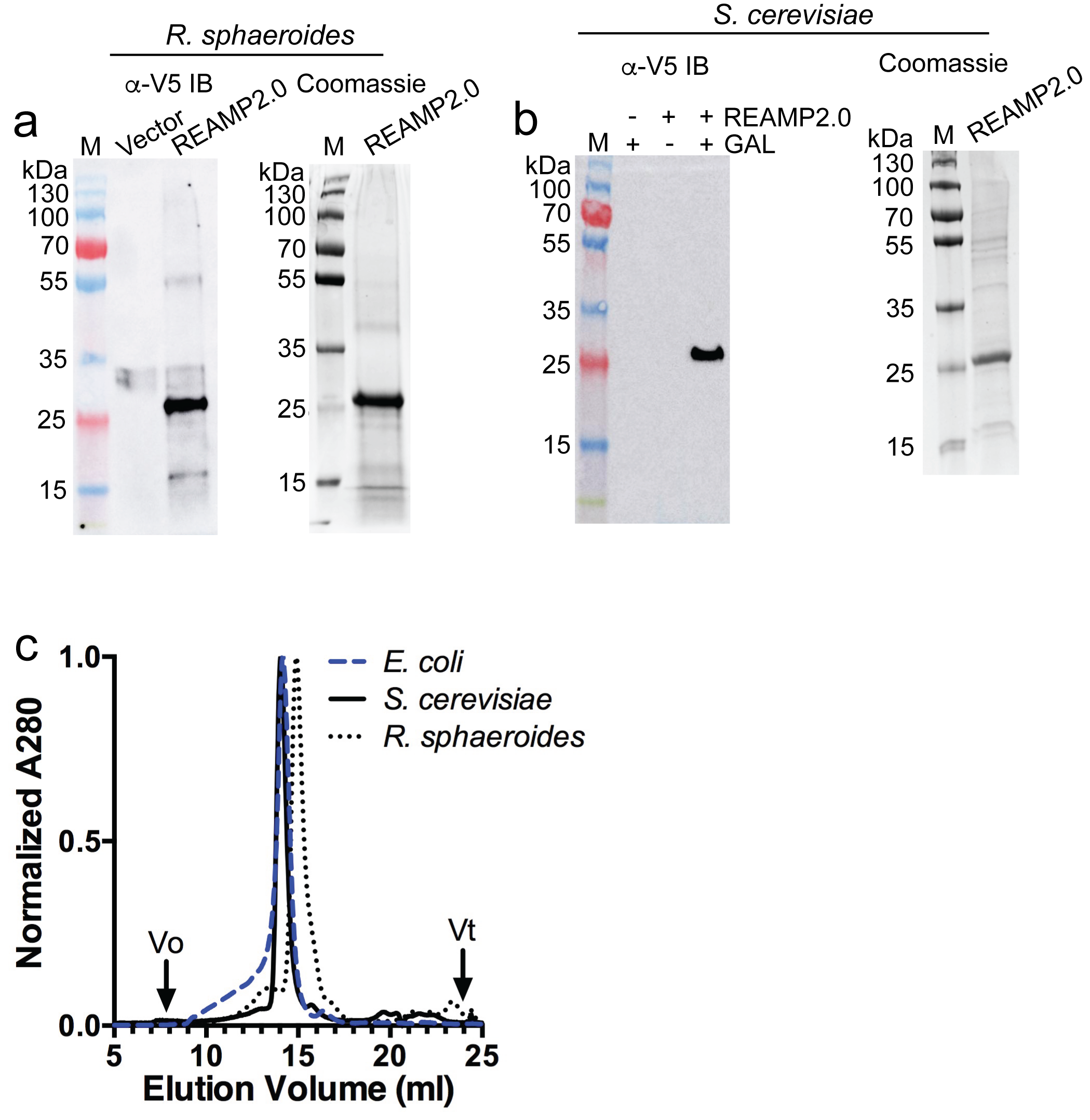


Figure S4. REAMP2.0 can be expressed in diverse recombinant hosts. (**a**) Expression in the purple photosynthetic bacterium *Rhodobacter sphaeroides* is confirmed by western blotting against the V5 epitope (*α-V5-IB)* versus an empty vector control. After purification from cell membranes, a single major band corresponding to REAMP2.0 is observed by staining with Coomassie Brilliant Blue. (**b**) Expression in the yeast S*accharomyces cerevisiae* from a galactose-inducible promoter is confirmed by western blotting as above. A band corresponding to REAMP2.0 is evident in recombinant strains in the presence of galactose (*GAL*), and not in uninduced or vector-only controls. After purification from cell membranes, a single major band corresponding to REAMP2.0 is observed by Coomassie staining. (**c**) The purified proteins all run as a single species on size exclusion chromatography in the presence of the detergent Cymal-5.

**SUPPLEMENTARY FIGURE S5**

**
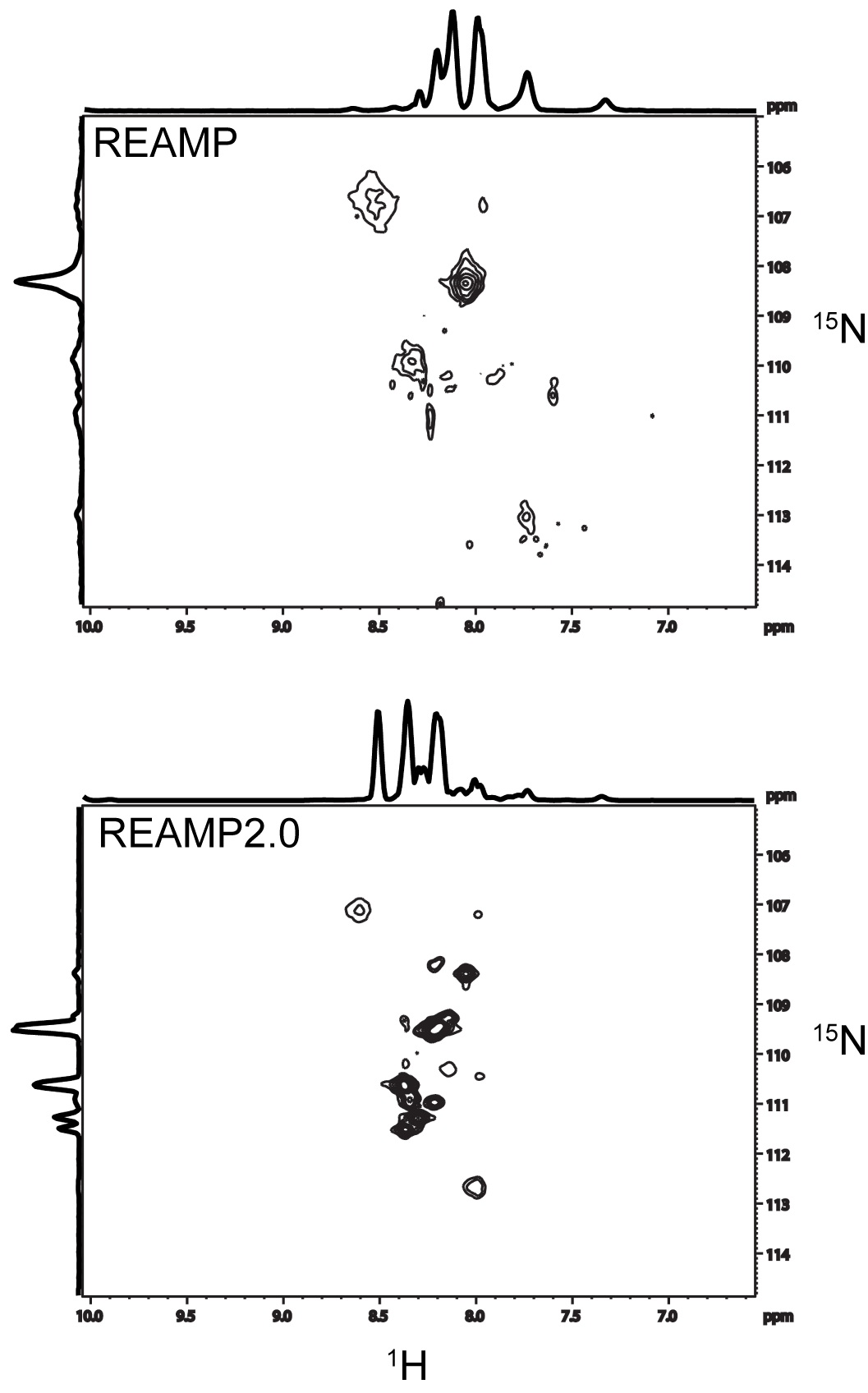
**

**Figure S5.** Close-up of the glycine region of ^1^H-^15^N HSQC NMR. Several new peaks emerge in the spectrum of REAMP2.0, consistent with a change in environment around those residues.

**SUPPLEMENTARY FIGURE S6**


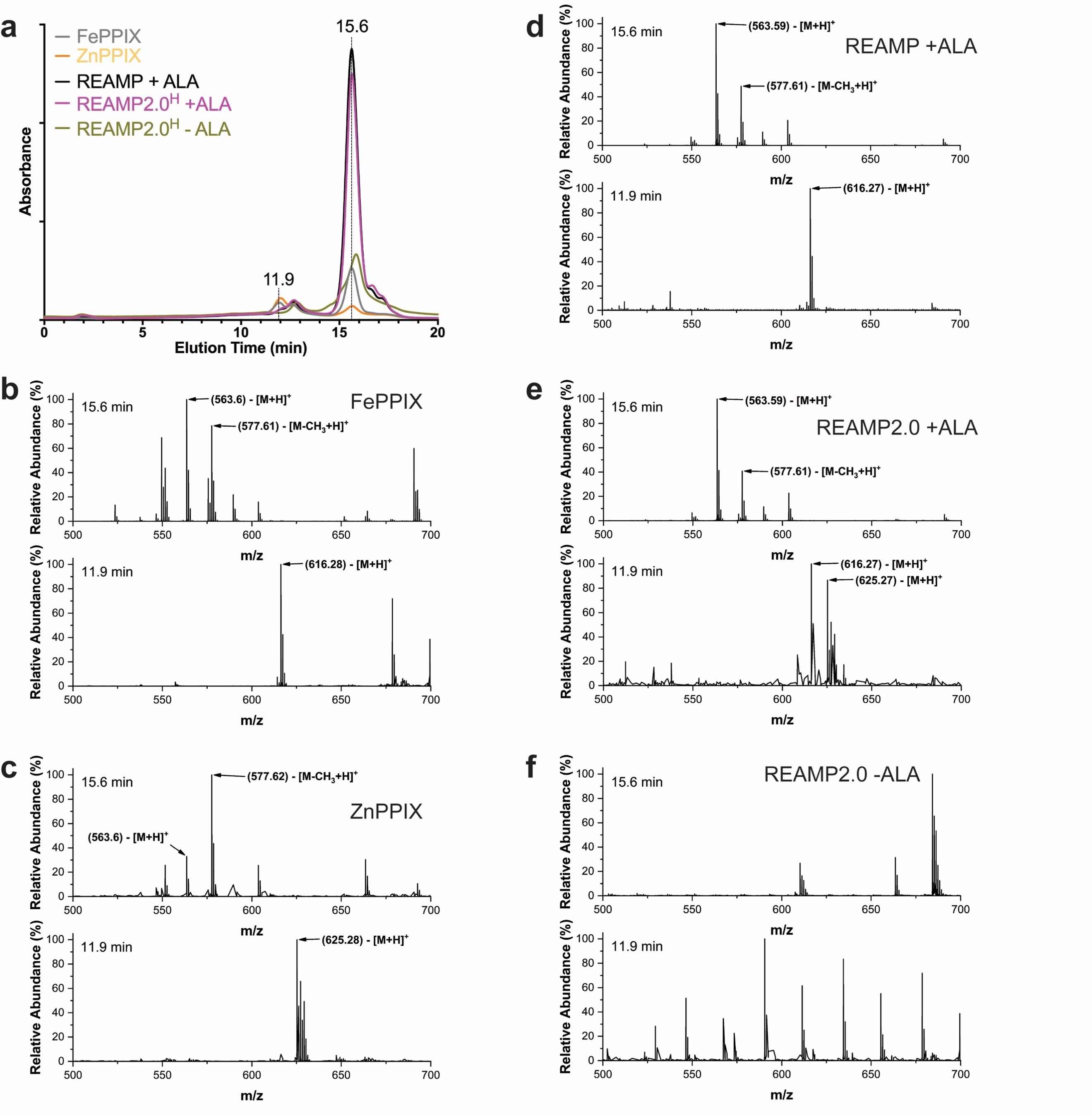


Figure S6. LC-MS analysis confirms the pigment produced by REAMP2.0 strains of *E.coli* is ZnPPIX. (**a**) LC of porphyrin standards and cell membrane extracts. Standards of (**b**) FePPIX and (**c**) ZnPPIX elute as the metallated form at 11.9 min and as the demetallated (free base) porphyrin at 15.6 min. (**d**) Membrane extract from cells expressing REAMP and supplemented with *δ*-aminolevulinic acid (ALA). (**e**) Equivalent membranes from supplemented cells expressing REAMP2.0 have the same porphyrin profile but also accumulate ZnPPIX. (**f**) Control membranes from unsupplemented cells contain negligible porphyrin. Theoretical weights are: PPIX, 562.7 g/mol; FePPIX, 616.5 g/mol; ZnPPIX, 626.0 g/mol. Peak at m/z=577.6 is likely from acid-catalyzed Fisher esterification of the heme propionate and methanol. We did not resolve other porphyrins under the conditions used.

**SUPPLEMENTARY FIGURE S7**


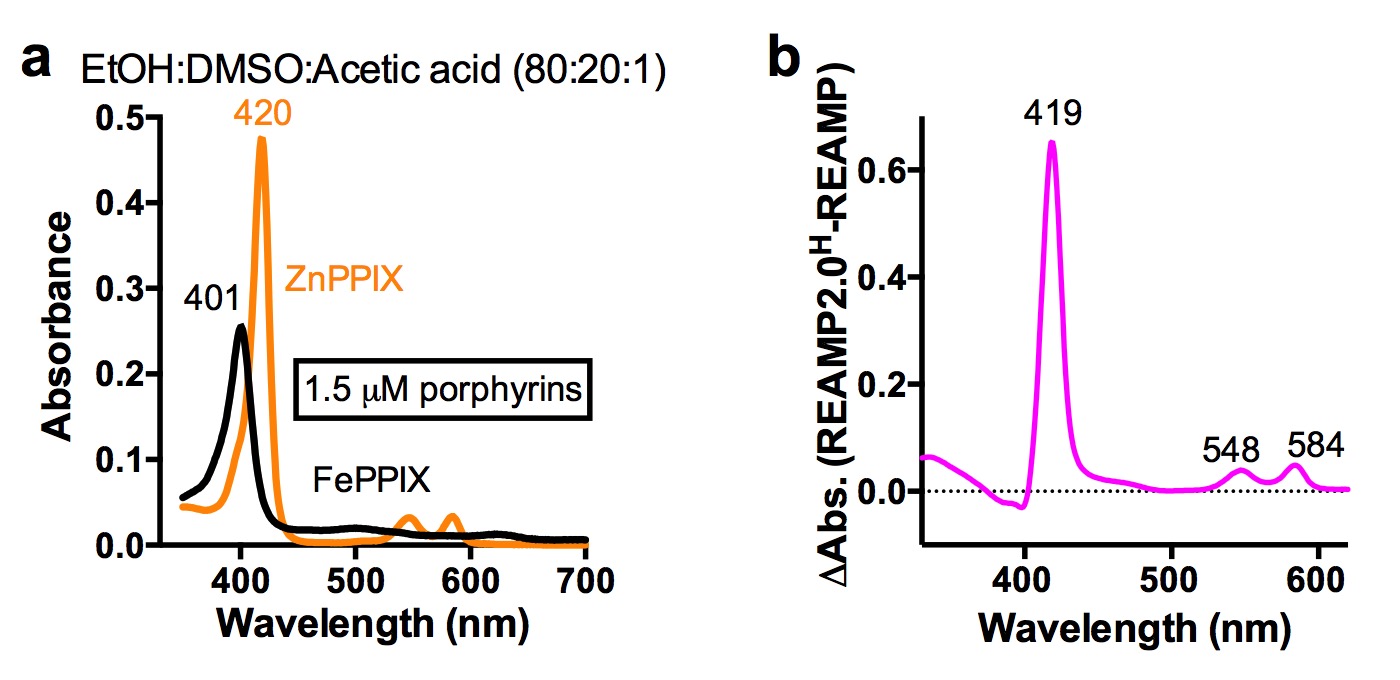


Figure S7. (**a**) Absorption spectra of commercial standards of zinc and iron protoporphyrin IX in the membrane extract solvent used in this study (80:20:1 v/v Ethanol:DMSO:acetic acid). (**b**) Difference spectrum of membrane extracts from ALA-supplemented strains induced to express either REAMP2.0^H^ or REAMP. The content of heme is approximately the same in both strains (ΔAbs401 = 0).

**SUPPLEMENTARY FIGURE S8**


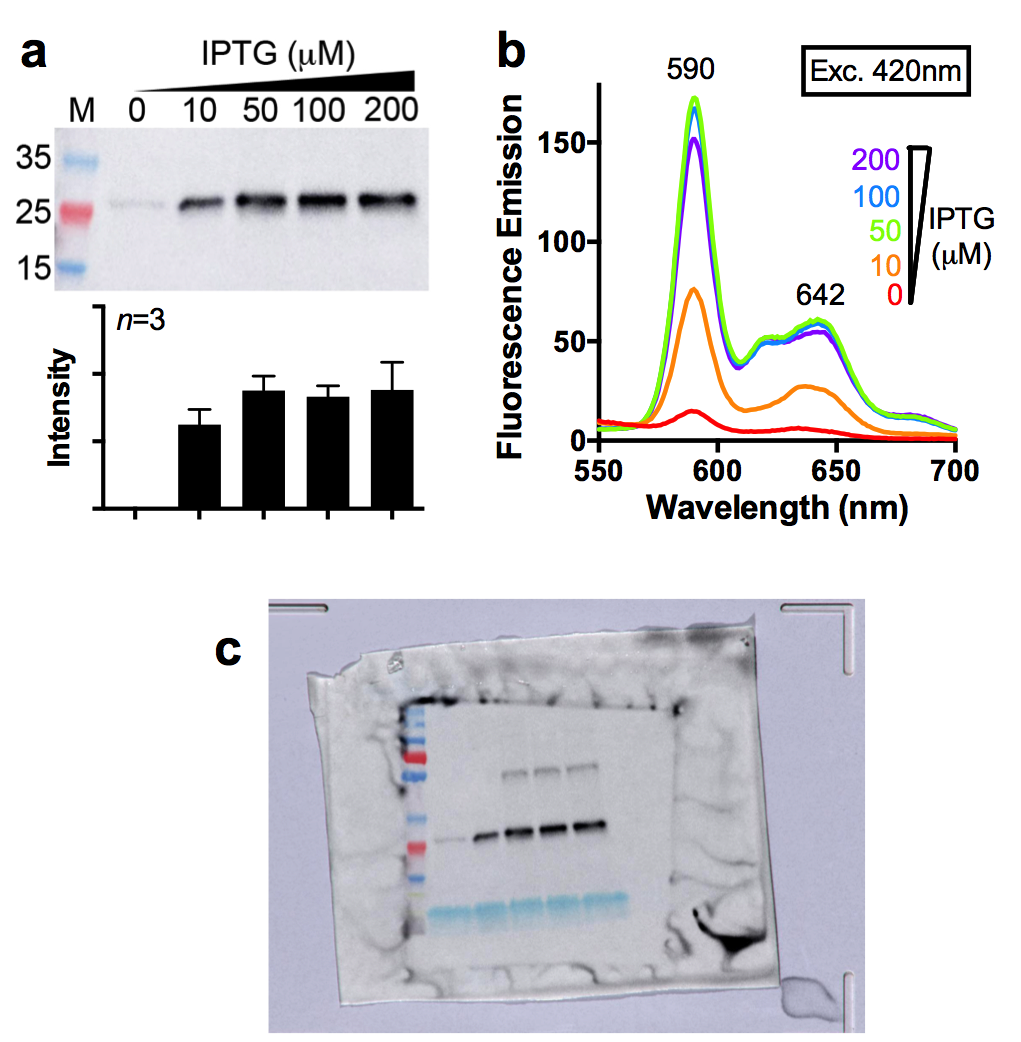


Figure S8. REAMP2.0^H^ expression and zinc porphyrin production monitored in whole cell lysates after treatment with BugBuster™ reagent. (**a**) Representative western blot (α-V5) of REAMP2.0^H^ expression at different inducer concentrations. Lower panel shows results of gel densitometry, mean ± SD of three independent replicates. (**b**) Fluorescence spectroscopy of the same cell extracts used in panel (**a**). (**c**) Unprocessed raw data underpinning panel (**a**). The image shows the same western blot as-recorded on an Amersham Imager (GE Healthcare). The PageRuler Plus prestained marker is used (Thermo Scientific).

**SUPPLEMENTARY FIGURE S9**


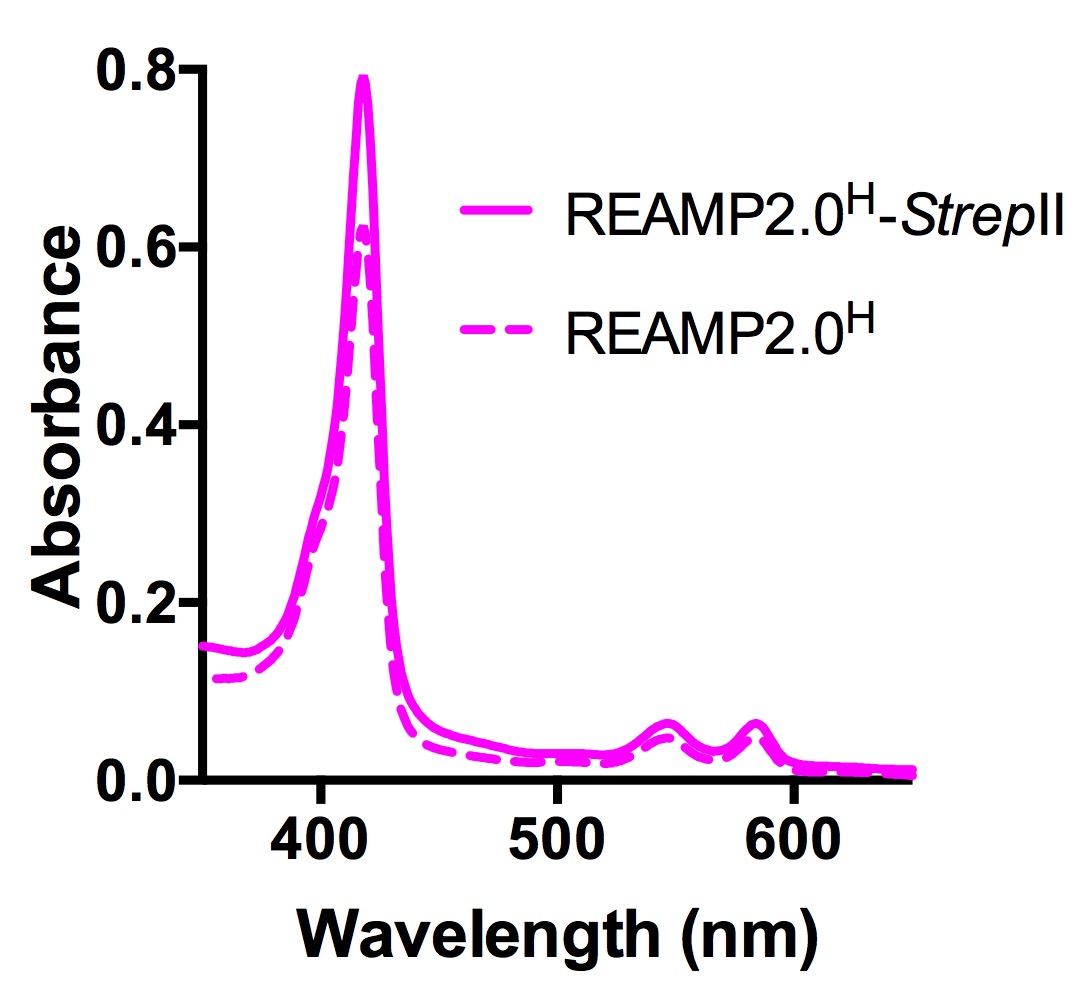


Figure S9. Solvent extracts of cellular membranes demonstrate that the StrepII purification tag is irrelevant to the accumulation of ZnPPIX in *E. coli* membranes. Data are not corrected for any variability in protein expression between strains.

**SUPPLEMENTARY FIGURE S10**


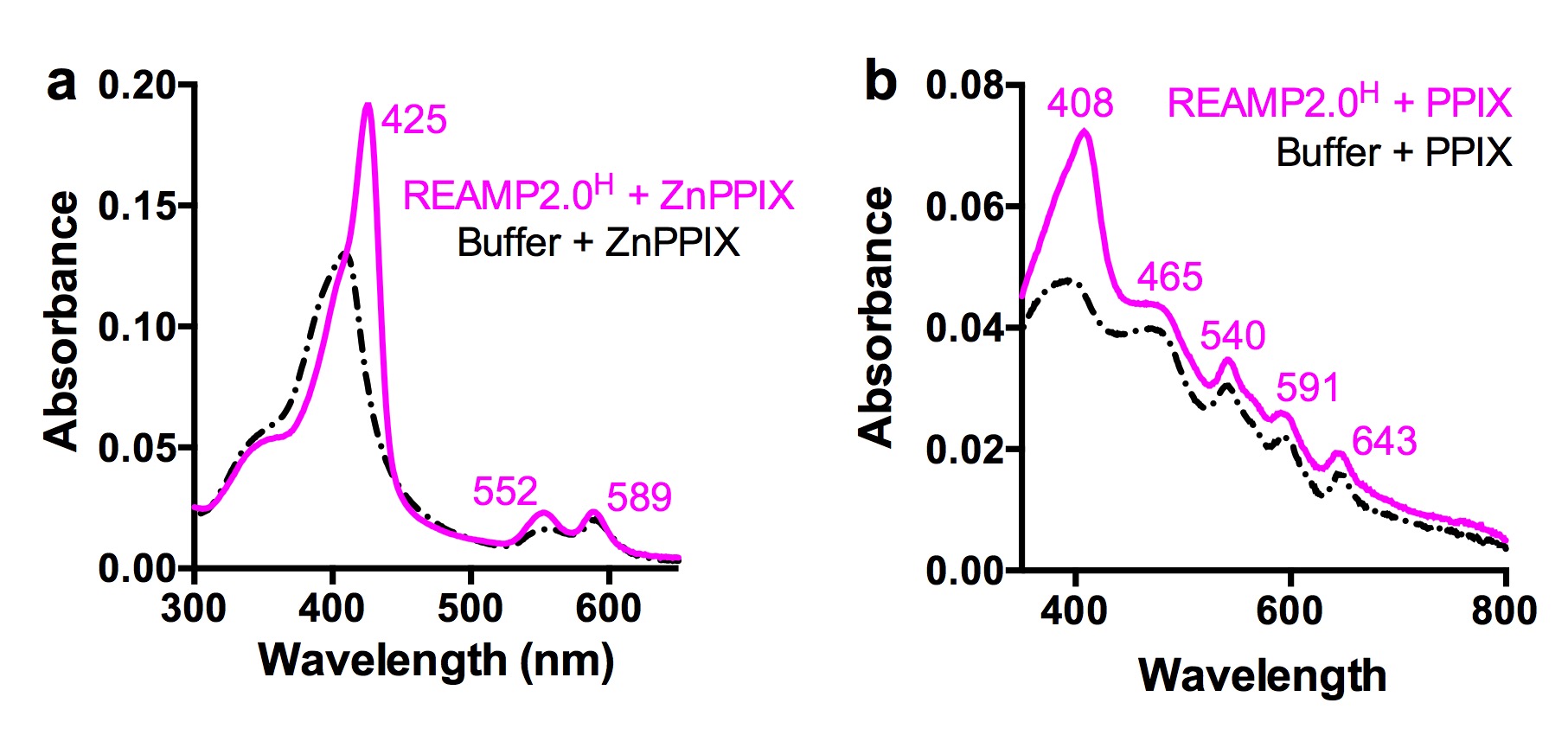


Figure S10. Purified REAMP2.0^H^ co-ordinates ZnPPIX and demetallated protoporphyrin IX (PPIX) *in vitro*. Data collected with both protein and cofactor at 1.5 μM.

**SUPPLEMENTARY FIGURE S11**


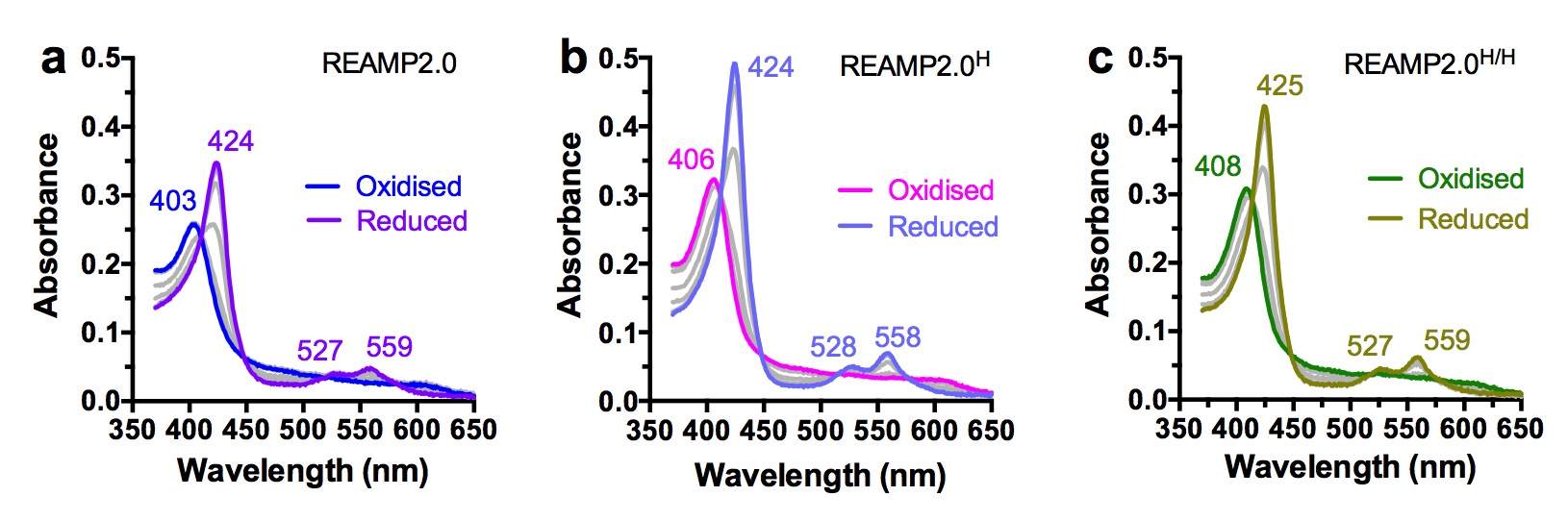


Figure S11. Potentiometric redox titrations of (**a**) REAMP2.0, (**b**) REAMP2.0^H^ and (**c**) REAMP2.0^H/H^ .

**SUPPLEMENTARY FIGURE S12**


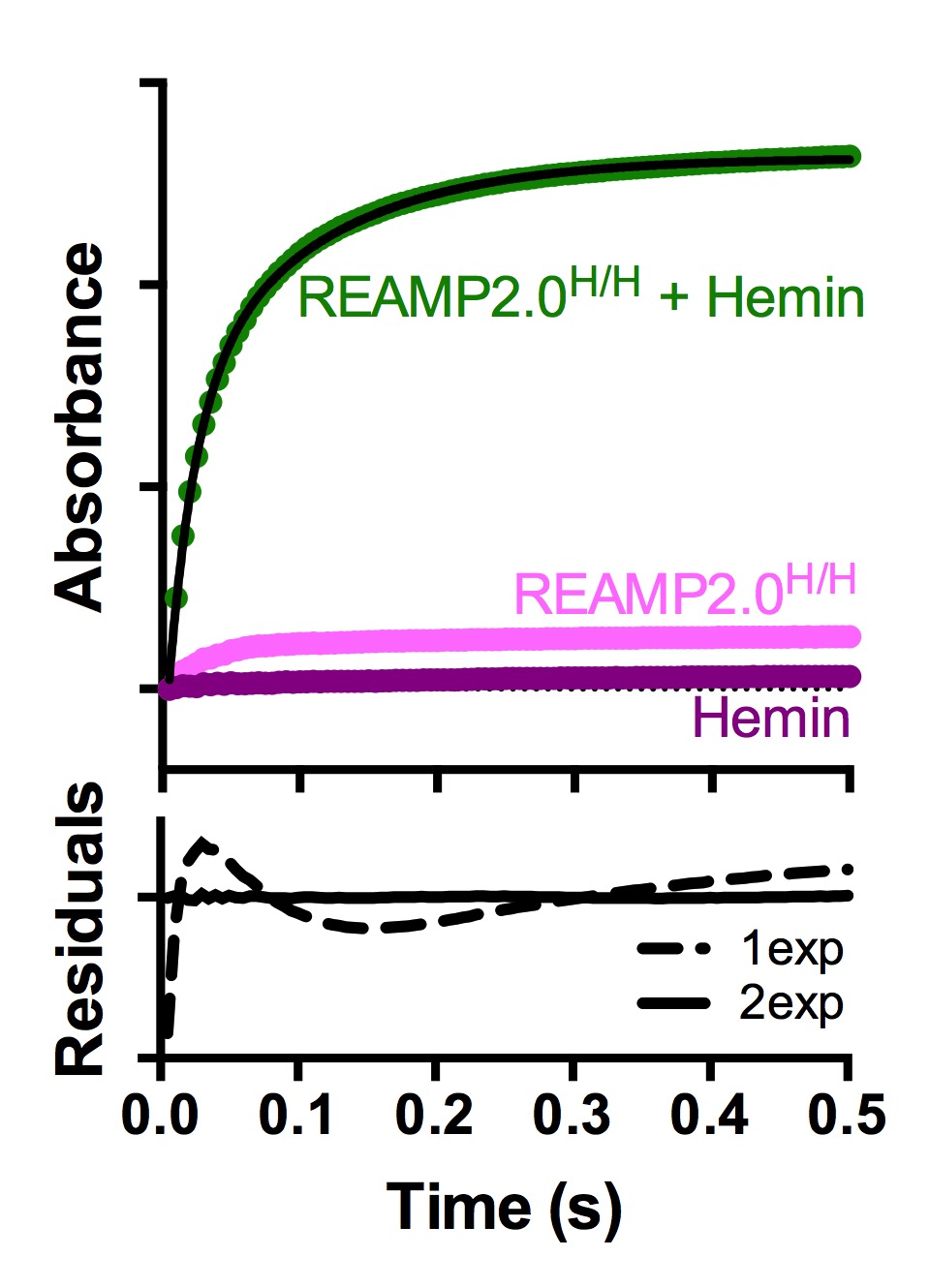


Figure S12. Heme binding to REAMP2.0^H/H^ is best fit to the sum of two exponents.

**SUPPLEMENTARY FIGURE S13**


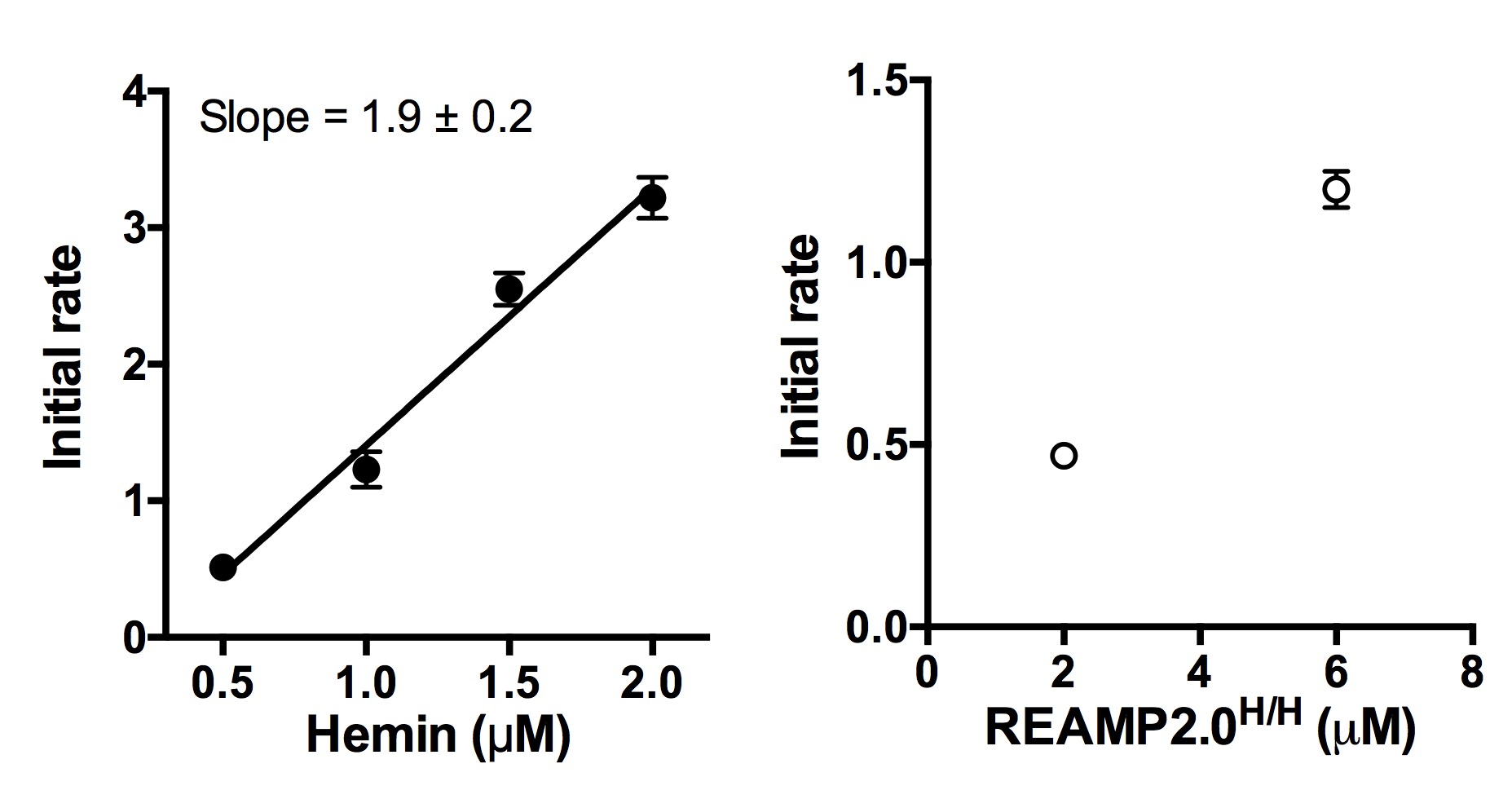


Figure S13. The initial (linear) rate constant of heme binding is first-order with respect to both heme and protein concentration, characteristic of a second-order bimolecular reaction.

**SUPPLEMENTARY FIGURE S14**


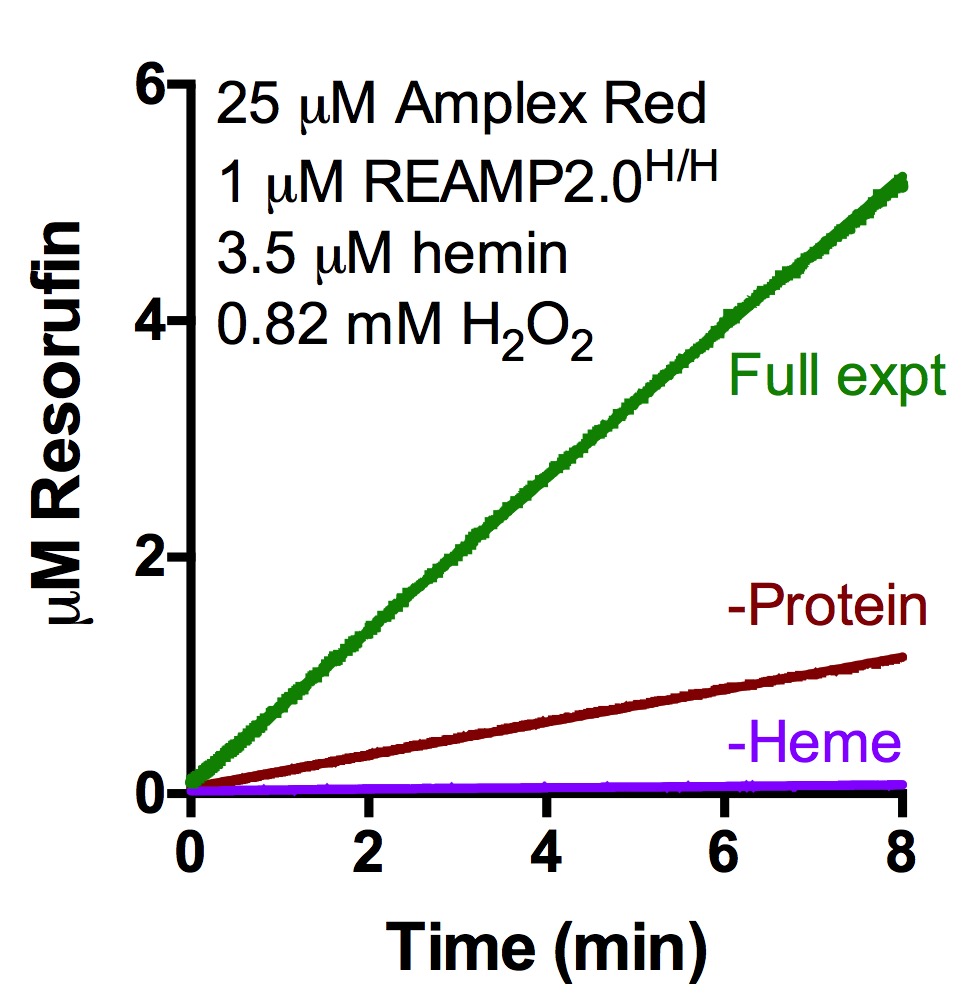


Figure S14. Activity of REAMP2.0^H/H^ towards the peroxidase substrate Amplex Red. The initial rate under these conditions is indistinguishable from that recorded with ABTS.

**SUPPLEMENTARY METHODS**

*Expression in different recombinant hosts*

To express REAMP2.0 in *R. sphaeroides* and *S. cerevisiae*, the *Strep*II-tagged sequence was amplified by PCR to introduce either NcoI/NgoMIV or HindIII/XbaI restriction sites at either end of the gene. Expression in *R. sphaeroides* used plasmid pv39, a derivative of pRK415 with an insert that encompasses the *pufBA* genes of the *sphaeroides* light-harvesting antenna complex. The *pufBA* genes were excised from this insert with NcoI/NgoMIV and replaced with REAMP2.0 via cohesive-end ligation. This places the expression of REAMP2.0 under control of the *puf* operon promoter. *R.* sphaeroides was cultured in M22+ media under dark/semiaerobic conditions as described previously^8^. For *S. cerevisiae* the insert was ligated into the shuttle vector pYES2/CT (ThermoFisher) using cohesive end ligation with HindIII/XbaI before expression in the protease-deficient auxotroph strain FGY217^9^. Yeast cultures were grown overnight in Ura^-^ media with 2% glucose. These were diluted to A600 = 0.1 in Ura^-^ media containing 2% galactose/0.1% glucose and cultured for 24h to allow expression from the GAL promoter.

*Mass spectrometry*

Extracted porphyrins were analysed by liquid chromatography-mass spectrometry using a C8 reverse column (Grace Vydac, 100 x 21 mm, 5 μm) with a two solvent 20-minute gradient mobile phase (Solvent A = 0.5M ammonium acetate:CH_3_CN, 90:10 vol/vol pH 5.1; solvent B = 90:10% MeOH:CH_3_CN; gradient was 95:5 A:B to 5:95 A:B, 0.25 ml/min). The injection volume was 20 μl and the chromatogram was determined by absorbance at 254 nm. The column eluent entered an isocratic solvent chamber and was diluted 1:100 prior to positive electron-spray-ionization (ESI) mass spectrometry (Waters Xevo G2-XS QTof). The mass spectrum contained peaks screened across a m/z range of 400-700.

*Cell imaging*

For light microscopy, bacteria were fixed in 2% w/v paraformaldehyde in PBS for 20 minutes followed by a further 3 washes in PBS. 10 µl of the cell suspension was mounted between a glass slide and coverslip in ProLong Gold antifade (ThermoFisher). Images were acquired on a Leica SP8 AOBS confocal laser scanning microscope attached to a Leica DMi8 inverted epifluorescence microscope (Leica Microsystems). Images were acquired using a 100x 1.4 NA oil immersion objective.

For electron microscopy, 1μl of cells were loaded into a 0.1 mm membrane carrier (Leica) and vitrified by high pressure freezing (EMPACT2, Leica). Frozen membrane carriers were freeze-substituted in an automated freeze-substitution unit (AFS2, Leica) in 1% osmium tetroxide, 0.1% uranyl acetate in anhydrous acetone^10^ or in 0.2% uranyl acetate, 5% water in anhydrous acetone^11^ before embedding in EPON or HM20 resin. Polymerised blocks were sectioned with a 45° diamond knife using an EM UC6 microtome (Leica). 70 nm thick sections were collected on copper slot grids (Agar Scientific) coated in carbon and pioloform. Grids were post-stained with 3% uranyl acetate and Reynolds lead citrate for 10 and 4 min respectively. Images were collected using a Tecnai 12 120kV BioTwin Spirit transmission electron microscope equipped with an FEI Ceta 4k x 4k CCD camera operated at an accelerating voltage of 120 kV.

**REFERENCES**

1 Tsirigos, K. D., Peters, C., Shu, N., Käll, L. & Elofsson, A. The TOPCONS web server for combined membrane protein topology and signal peptide prediction. *Nucleic Acids Res* **43**, W401-W407 (2015).

2 Krogh, A., Larsson, B., von Heijne, G. & Sonnhammer, E. L. L. Predicting transmembrane protein topology with a hidden markov model: application to complete genomes. *Journal of molecular biology* **305**, 567-580 (2001).

3 Tusnády, G. & Simon, I. The HMMTOP transmembrane topology prediction server. *Bioinformatics* **17**, 849-850 (2001).

4 Peters, C., Tsirigos, K. D., Shu, N. & Elofsson, A. Improved topology predictions using the first and last hydrophobic helix rule. *Bioinformatics* **32**, 1158-1162 (2015).

5 Reynolds, S. M., Käll, L., Riffle, M. E., Bilmes, J. A. & Noble, W. S. Transmembrane topology and signal peptide prediction using dynamic bayesian networks. *PLoS Comput Biol* **4**, e1000213 (2008).

6 Lalaurie, C. J. *et al.* The *de novo* design of a biocompatible and functional integral membrane protein using minimal sequence complexity. *Scientific Reports* **8**, 14564 (2018).

7 Slotboom, D., Duurkens, R., Olieman, K. & Erkens, G. Static light scattering to characterize membrane proteins in detergent solution. *Methods* **46**, 73-82 (2008).

8 Swainsbury, D. J. K., Friebe, V. M., Frese, R. N. & Jones, M. R. Evaluation of a biohybrid photoelectrochemical cell employing the purple bacterial reaction centre as a biosensor for herbicides. *Biosensors and Bioelectronics* **58**, 172-178 (2014).

9 Kota, J., Gilstring, C. F. & Ljungdahl, P. O. Membrane chaperone Shr3 assists in folding amino acid permeases preventing precocious ERAD *J. Cell Biol.* **176**, 617-628 (2007).

10 Verkade, P. Moving EM: the Rapid Transfer system as a new tool for correlative light and electron microscopy and high throughput for high-pressure freezing. *J Microscopy* **230**, 317-328 (2008).

11 Lee, M. J. *et al.* Engineered synthetic scaffolds for organizing proteins within the bacterial cytoplasm. *Nat Chem Biol* **14**, 142-147 (2018).
