# Supplementary material for: Small-residue packing motifs modulate the structure and function of a minimal de novo membrane protein
Source: Sci Rep. 2020 Sep 16;10:15203. doi: 10.1038/s41598-020-71585-8 (PMC7495484; doi:10.1038/s41598-020-71585-8)
Supplement: Supplementary file 2 — Supplementary file2 [file 41598_2020_71585_MOESM2_ESM.zip › Modelling/Modelling supplement Curnow et al 2020.docx]

This zip folder contains the transformed PDB files, span files, flags and protocols used for packing with RosettaMP implemented in Rosetta release 2018.33. These were run on the University of Bristol high-performance computing cluster BlueCrystal4.

**Transformed input files**

Files labelled as “transformed” are PDB files for the input structures, transformed into membrane coordinates with the PPM server (<https://opm.phar.umich.edu/ppm_server>) and mp_transform as follows.

PDB file output from the PPM server was used to generate spanfiles using:

$ mp_span_from_pdb.linuxgccrelease -in:file:s <*filename*>.pdb

Before the transformed structure was generated with:

$ mp_transform.linuxgccrelease -in:file:s <*filename*>.pdb \

-mp:setup:spanfiles <*filename*>.span

**Packing simulation**

Packing of the entire protein constructs (including loops and C-terminal tags) was performed with a Rosetta script termed membrane_relax.xml as follows:

<ROSETTASCRIPTS>

<SCOREFXNS>

<ScoreFunction name="memb_hires" weights="mpframework_smooth_fa_2012"/>

</SCOREFXNS>

<TASKOPERATIONS>

<ExtraRotamersGeneric name="extra_rot" ex1="1" ex2="1"/>

<InitializeFromCommandline name="ifcl"/>

</TASKOPERATIONS>

<MOVERS>

<AddMembraneMover name="add_memb"/>

<MembranePositionFromTopologyMover name="init_pos"/>

<FastRelax name="fast_relax" scorefxn="memb_hires" repeats="8" task_operations="ifcl,extra_rot"/>

</MOVERS>

<PROTOCOLS>

<Add mover="add_memb"/>

<Add mover="init_pos"/>

<Add mover="fast_relax"/>

</PROTOCOLS>

<OUTPUT scorefxn="memb_hires" />

</ROSETTASCRIPTS>

**Using the following flags:**

-parser:protocol membrane_relax.xml

-in:file:s reamp2_transformed_relaxed_cst_4.pdb

-in:file:native reamp2_transformed_relaxed_cst_4.pdb

-mp:scoring:hbond true

-mp:setup:spanfiles reamp2.span

-ignore_unrecognized_res

-use_input_sc

-nstruct 500

-relax:jump_move true

-out:file:scorefile scores.sc

-packing:pack_missing_sidechains 0

Jobs were submitted using Slurm with the command line:

$ sbatch relax_slurm.sh

With the corresponding Slurm file:

#!/bin/bash

#SBATCH --job-name=rlx_R2pt0_10000

#SBATCH --partition=serial

#SBATCH --nodes=1

#SBATCH --ntasks-per-node=1

#SBATCH --cpus-per-task=1

#SBATCH --time=3-0:0:00

#SBATCH --mem=5GB

#SBATCH --array=1-20

srun rosetta_scripts.static.linuxgccrelease @relax_10000_flags \

-out:suffix $SLURM_ARRAY_TASK_ID \

-out:prefix $SLURM_JOBID \

-out:file:silent struct_500_$SLURM_ARRAY_TASK_ID \

-out:path:all output_10000 \

**Analysis**

For comparative analysis the transmembrane (TM) domains were re-scored. The top-scoring decoy was then used as the reference for calculating RMSD. The relevant script was termed **rescore_rmsd.xml** and included a filter. The following example shows the script used for REAMP2.0:

<ROSETTASCRIPTS>

<SCOREFXNS>

<ScoreFunction name="memb_hires" weights="mpframework_smooth_fa_2012"/>

</SCOREFXNS>

<RESIDUE_SELECTORS>

<Index name="tm1" resnums="3-22"/>

<Index name="tm2" resnums="51-69"/>

<Index name="tm3" resnums="100-116"/>

<Index name="tm4" resnums="147-166"/>

<Or name="TM" selectors="tm1,tm2,tm3,tm4"/>

</RESIDUE_SELECTORS>

<FILTERS>

<RmsdFromResidueSelectorFilter name="TM_RMSD" superimpose="1" confidence="0" reference_selector="TM" query_selector="TM"/>

<ScorePoseSegmentFromResidueSelectorFilter name="TM_score" in_context="1" residue_selector="TM" scorefxn="memb_hires" confidence="0"/>

</FILTERS>

<MOVERS>

<AddMembraneMover name="add_memb"/>

<MembranePositionFromTopologyMover name="init_pos"/>

</MOVERS>

<PROTOCOLS>

<Add mover_name="add_memb"/>

<Add mover_name="init_pos"/>

<Add filter_name="TM_RMSD"/>

<Add filter_name="TM_score"/>

</PROTOCOLS>

</ROSETTASCRIPTS>

Where TM domains were taken from the relevant span files for REAMP2 and REAMP.

The following command was used to execute this script:

$ rosetta_scripts.linuxgccrelease -parser:protocol rescore_rmsd.xml -in:file:silent struct_500_* -in:file:native <*lowest_scoring_filename*>.pdb -mp:setup:spanfiles reamp2.span -mp:scoring:hbond -out:file:scorefile rescored_rmsd_tm_all.sc -out:file:silent rescoring_rmsd_all

Where “-in:file:silent struct_500_*” samples each of the 20 silent files generated by the original simulation.
